# Electricity and Water Conservation on College and University Campuses in Response to National Competitions among Dormitories: Quantifying Relationships between Behavior, Conservation Strategies and Psychological Metrics

**DOI:** 10.1371/journal.pone.0144070

**Published:** 2015-12-16

**Authors:** John E. Petersen, Cynthia M. Frantz, Md. Rumi Shammin, Tess M. Yanisch, Evan Tincknell, Noel Myers

**Affiliations:** 1 Environmental Studies Program, Oberlin College, Oberlin, Ohio, United States of America; 2 Psychology Department, Oberlin College, Oberlin, Ohio, United States of America; 3 Applied Psychology Department, New York University—Steinhardt, New York, New York, United States of America; University of St Andrews, UNITED KINGDOM

## Abstract

“Campus Conservation Nationals” (CCN) is a recurring, nation-wide electricity and water-use reduction competition among dormitories on college campuses. We conducted a two year empirical study of the competition’s effects on resource consumption and the relationship between conservation, use of web technology and various psychological measures. Significant reductions in electricity and water use occurred during the two CCN competitions examined (n = 105,000 and 197,000 participating dorm residents respectively). In 2010, overall reductions during the competition were 4% for electricity and 6% for water. The top 10% of dorms achieved 28% and 36% reductions in electricity and water respectively. Participation was larger in 2012 and reductions were slightly smaller (i.e. 3% electricity). The fact that no seasonal pattern in electricity use was evident during non-competition periods suggests that results are attributable to the competition. Post competition resource use data collected in 2012 indicates that conservation behavior was sustained beyond the competition. Surveys were used to assess psychological and behavioral responses (n = 2,900 and 2,600 in 2010 and 2012 respectively). Electricity reductions were significantly correlated with: web visitation, specific conservation behaviors, awareness of the competition, motivation and sense of empowerment. However, participants were significantly more motivated than empowered. Perceived benefits of conservation were skewed towards global and future concerns while perceived barriers tended to be local. Results also suggest that competitions may be useful for “preaching beyond the choir”–engaging those who might lack prior intrinsic or political motivation. Although college life is distinct, certain conclusions related to competitions, self-efficacy, and motivation and social norms likely extend to other residential settings.

## Background

### 1.1. Buildings, resource use, and behavior-based competitions in college dormitories

North Americans spend more than 90% of their lives in buildings [1]. The built environment in which we live and work accounts for 41% of all energy consumption and 40% of greenhouse gas emissions [2] and 12% of fresh water consumption in the U.S. [3]. Buildings in the education sector consume about 11% of primary energy consumed by all commercial buildings in the U.S. [2]. On residential college and university campuses, activities that take place in buildings typically account for the vast majority of energy use, water use and total greenhouse gas emissions (e.g. >90% of total scope 1 and 2 emissions at Oberlin College, [4]). While schools and other organizations often focus on infrastructure renovation as a primary mechanism for increasing resource use efficiency, occupant behavior is recognized as a key determinant of consumption [5] and therefore a crucial target of conservation efforts (e.g., [6]). The emphasis on achieving behavioral change in colleges and universities also recognizes the undergraduate experience as a seminal and transformative period during which future decision-makers develop knowledge and habits that inform the personal, professional and political choices that they make throughout the rest of their lives [7]. A national competition among residence halls (in this paper referred to as dormitories or “dorms”) on college and university campuses was proposed as a mechanism for developing and encouraging behavioral change and community engagement related to resource conservation [8]. The first “Campus Conservation Nationals” (CCN) was held in fall of 2010 with 39participating schools. The CCN competition has since been repeated in spring of 2012, 2013 and 2014with a trend of increasing numbers in subsequent years. This paper provides a detailed analysis of the first two CCN events to assess the efficacy of competitions as means of changing thought and behavior and to better understand the psychological factors involved.

### 1.2. Psychological basis for competition combined with feedback as a motivator

In considering why people choose to exhibit particular behaviors, research indicates that the impact of money, other incentives and knowledge is often overestimated (e.g., [9]) while people’s perceptions of what other people are doing (i.e. the impact of social norms) is often underestimated (e.g., [10]). Indeed, in regards to encouraging resource use reductions, normative social influence has been shown to be significantly more motivating in stimulating energy conservation behavior than environmental, financial or societal benefits [10]. Structured competitions, one form of social comparison, provide a potentially powerful mechanism for leveraging the power of social norms [11].

Competition must achieve four related goals in order to stimulate change in thought and behavior that result in short and long-term resource-use reduction [12]. The competition must be structured to:

1. engage (catch attention of and involve the target audience),
2. educate (communicate information on what, why and how behavior should change),
3. motivate (enhance desire to change behavior) and
4. empower (increase perception and reality of self-efficacy and suggest concrete and actionable behavior).

These goals are closely aligned with a variety of psychological models including the theory of planned behavior [13].

Effective competition is contingent on delivery of salient information regarding both absolute and comparative performance. “Sociotechnical” feedback is a special class of information feedback in which technology is employed to acquire, process and deliver content that alters human thought and action. There are multiple ways in which feedback on resource consumption has proved an effective tool for motivating conservation [12]. Prior research demonstrates that socially and environmentally contextualized feedback on resource use combined with competition among dorms can result in significant short-term reductions in resource use in individual schools [7]. For example, reductions in electricity use up to 56% have been documented in best performing dorms during resource reduction competitions that included education and real-time feedback [7].

While most studies examining the impact of feedback have focused on individual households, there is data from a variety of sources that suggest that larger groups such as offices and dorms can conserve resources in response to comparisons of aggregated use (e.g., [14]). Organizers of the CCN speculated that expanding the scale of a feedback–so that it included comparison, competition and/or collaboration among schools as well as competition among dorms within each school–might provide a mechanism for promoting participation and enhancing national and local impact [8]. Rivalries among dorms within schools are paralleled by rivalries among schools that are perceived to be peer institutions. Perception of peer status at the institutional level is based on a variety of distinct attributes that may include overlapping applicant pools, degree of admission selectivity, shared athletic conferences, public vs. private, geographic proximity, institution size, etc.

### 1.3. Prior resource reduction competitions at schools and what they tell us

Annual resource-use reduction competitions have become increasingly popular at schools in the U.S. since the early 1990s. For example, Harvard University’s “Do it in the Dark” competitions, one of the earliest we are aware of, focused on electricity use reduction in dormitories. Duke University’s “Ecolympics”, in contrast, has combined conservation competitions among dorms with a range of other sustainability-related competitive events. An analysis of data obtained from the Sustainable Endowments Institute’s 2010 survey of schools revealed that 163 colleges and universities in the U.S. and Canada had run or intended to run competitions [15]. A recent case study considered lessons learned from 20 distinct energy reduction competitions occurring in a broad range of contexts including colleges and universities, non-academic residential environments and commercial settings [11]. In recognition of the growing prevalence of these competitions, the Association for the Advancement of Sustainability in Higher Education posted the document “Organizing residence hall conservation competitions, a guide for students” [16] that provides examples and advice for schools interested in organizing competitions.

In addition to within-school competitions, a limited number of national-level resource-use reduction competitions have been attempted on college campuses with different degrees of success. These efforts have varied in a number of important respects including: 1) resources considered (e.g. material waste, electricity, total energy, greenhouse gas emissions and water); 2) type of buildings included (e.g. dormitories vs. academic buildings); 3) degree of aggregation and scale (e.g. competitions among individual buildings vs. competitions among entire campuses); and 4) the way in which performance is quantified and provided as feedback to participants (e.g. automated real-time feedback on websites vs. manually collected data posted on bulletin boards at the close of a competition).

An examination of three prior efforts at national competitions provides useful context for understanding the rationale and results of the CCN effort. “RecycleMania” is perhaps the best example of a successful recurring national resource-use reduction competition (www.recyclemaniacs.org). In this annual 8-week contest schools compete against other schools to achieve highest rates of per capita and total recycling and material waste reduction. First initiated in 2001, participation grew each year to a peak of 630 participating schools in 2011 (data reported on website above). During the competition, coordinators on each campus use a web form to report weekly updates that are then posted for all participating campuses to view.

Prior to CCN, efforts at national competitions within and among schools to reduce energy or electricity use and greenhouse gas emissions had not proved sustainable. For example, a “National Campus Energy Competition” initiated in 2008 under the auspices of the Energy Action Coalition (www.energyactioncoalition.org) attempted to organize schools to compete against each other to achieve the greatest percent reduction in *total* campus energy use during the month of February (relative to baseline data from February of 2006). This effort proved unsuccessful for a variety of reasons including the complexity of accounting for total energy use, lack of self-efficacy on the part of students, lack of performance feedback during the competition and lack of organizational support and staffing [8]. “America’s Greenest Campus,” a national campus greenhouse gas reduction competition organized by SmartPower in 2009, serves as an interesting counterpoint to the National Campus Energy Competition. Institutions that participated in America’s Greenest Campus competed against each other based on self-reported commitments of individual students on each campus to change their behavior rather than on any direct measures of actual resource-use reduction. We are unaware of any previous national efforts that have included a significant focus on water. Of these three prior efforts, only RecycleMania was sustained beyond a single year.

### 1.4. Why focus competitions on electricity and water in residential housing?

The CCN competitions discussed in this paper were distinct from the three national competitions discussed above in their focus on electricity and water reductions in residence halls (dorms). A principle rationale for focusing on dorms is that this is the environment in which students exert the greatest degree of direct control over resource flows and it is therefore an environment in which they are most likely to be able to witness the effects of altering their behavior if feedback is provided [7]. It is also an environment in which students form personal and community habits that will remain with them throughout adulthood. Additionally, electricity and water use are often already monitored by utility meters so that the information necessary for feedback already exists in some form.

Energy (and sometimes water) used for heating, ventilation and air conditioning (HVAC) are important components of resource consumption in dorms. Indeed, more than 70% of primary energy used in educational buildings in the U.S. is used for HVAC and water heating [2]. Typically, natural gas, fuel oil and coal, often burned in central heating plants, are the dominant campus fuel source on campus. Although electrical energy is used for air conditioning and mechanical systems (and sometimes ground-source heat pumps), electrical energy use for HVAC is typically small relative to these other sources. In spite of its overall importance, non-electric HVAC energy was excluded from the CCN competition for three reasons. First, except in the still unusual circumstance of occupant adjustable thermostats, students’ capacity to control HVAC energy use is limited or entirely absent. Second, when campus-wide central HVAC plants are involved, consumption in individual dorms is often difficult to isolate and measure. Third, it is logical to assume that the magnitude and timing of energy used for HVAC loads varies considerably over geographic regions and by season making it difficult to isolate behavioral and to control for seasonal effects during a competition.

Within the resource conservation and advocacy literature, much emphasis is placed on the aggregate impact of changes in individual behavior (e.g. the proliferation of “100 simple things you can do to save the planet” type books). Others have pointed out the insufficiency of individual consumer-based action (e.g., [17]). CCN was framed by its organizers as an event that emphasized both individual and collective action and commitment at several levels–individual conservation commitments and actions by each student, collective commitments, competition among dormitories within each school, competitive dynamics among peer schools and collaborative dynamics nationally to achieve collective goals of resource use reduction. In addition to affecting beliefs, attitudes and consumptive behavior, organizers hoped that the competition could serve as a mechanism for building community and identity within and across campuses around issues of resource conservation and personal and group responsibility. A final goal was to use the competition as a mechanism for student organizers to develop leadership and community organizing skills that they might then apply at local, regional and national levels [8] (for current list of CCN goals see http://competetoreduce.org/ccn/about.html).

### 1.5. Theoretical context and research questions

A wide variety of theories and approaches can and have been used to explain factors responsible for behavior change (for review related to feedback interventions see [18]). Program evaluations such as the one described here pose both opportunities and challenges for assessing and advancing these models and approaches. An obvious advantage of program evaluation is that, assuming that change in thought and behavior can be systematically measured, the results provide a direct measure of the efficacy of a behavior change intervention. In the case of CCN, water and electricity use were directly measured in every participating dorm providing an excellent dataset for assessing actual changes in behavior in response to interventions associated with the program. A post-competition survey was used to capture a sample of self-reported changes in behavior and thought in response to students’ experience with this program. Minimally, these surveys provide the opportunity to assess self-perceptions of thought and behavior as well as the capacity to compare these self-perceptions with actual reductions and evaluate the veracity of relationships posited by theory.

The challenge of assessing a large-scale program such as the CCN is that diversity in behavioral manipulations and variability in execution on different campuses render it difficult to precisely test specific behavior change hypotheses. For example, the range of behavioral tools employed in CCN included: competition, cooperation, social norm stimulation, reciprocity, incentives, empathy stimulation, and appeals to concern for the environment. Topics of behaviour change research relevant to interpreting results therefore include the theory of planned behavior [13], connectedness with nature [19], community based social marketing [20], social norms (e.g., [21]), goal setting (e.g., [22]), commitment (e.g., [23]), peer networks, (e.g., [24]) and feedback (e.g., [14]). Because resource use behavior is determined by such a diverse array of influences we chose not to restrict ourselves to one particular theoretical lens (and currently no theory of behavior change incorporates all these elements). Rather CCN organizers leveraged a suite of behavior change tools in order to try to maximize impact, and those of us evaluating the program included measures of all the major psychological processes that previous empirical research has shown to be relevant. This design addresses the reality that different individuals experience different barriers and are motivated by different factors that may be addressed by different interventions [22, 25]. So, while a program evaluation may not be the ideal tool for precise hypothesis testing, a careful evaluation does provide a valuable opportunity for testing theories and approaches in a real world context.

#### 1.5.1. Goals and associated research questions

Six specific goals and associated questions posed and addressed in this study are outlined below.

Goal 1: Directly assess behavior changed in the form of resource use reductions in response to the competition.

1. Are there seasonal patterns of resource use that might confound interpretation of competition results?
2. Are reductions in electricity and water use during the competition statistically significant?
3. Are reductions sustained beyond the end of the competition?

Goal 2: Characterize website use and its relationship to reductions during competition

1. How extensively did students at competing schools use the competition website?
2. Is use of the website related to performance during the competition?
3. Are reductions related to the number of conservation commitments that students made on the website?
4. What types of feedback information and comparisons on the website were most used and valued by participants?
5. Does the presence of real-time feedback on the website result in greater conservation than less frequent feedback?

Goal 3: Characterize psychological variables related to resource use reductions and characterize relationships among these variables.

1. What changes in thought and behavior do participants report engaging in during the competition?
2. To what degree were competition participants, engaged, educated, motivated and/or empowered by the competition?
3. How powerful are social norms in motivating active participation in the competition?

Goal 4: Relate these psychological variables to resource use reduction.

1. Are changes in self-reported thought and behavior statistically linked to measured resource-use reductions during the competition?
2. Do demographic variables, political identity, environmental concern and degree of connectedness with nature influence performance in the competition?

Goal 5: Conduct additional analyses to further elucidate factors motivating behavior change and the efficacy of different strategies.

1. What do participants see as the principle barriers and benefits to participating in competitions?
2. How do participants differ in their thought and behavior with respect to water versus electricity competitions?
3. Is there evidence that competitions induce “spillover effects” in which conservation of one resource stimulate or enhance conservation of other resources?
4. Do participants differ in their response to the competition based on gender, ethnicity or political identity?

Goal 6: Identify factors that might make future behavioral interventions, including competitions on college and university campuses, more successful.

### 1.6. Competition Logistics

#### 1.6.1. Basics on competition and organization

**Fig 1:**
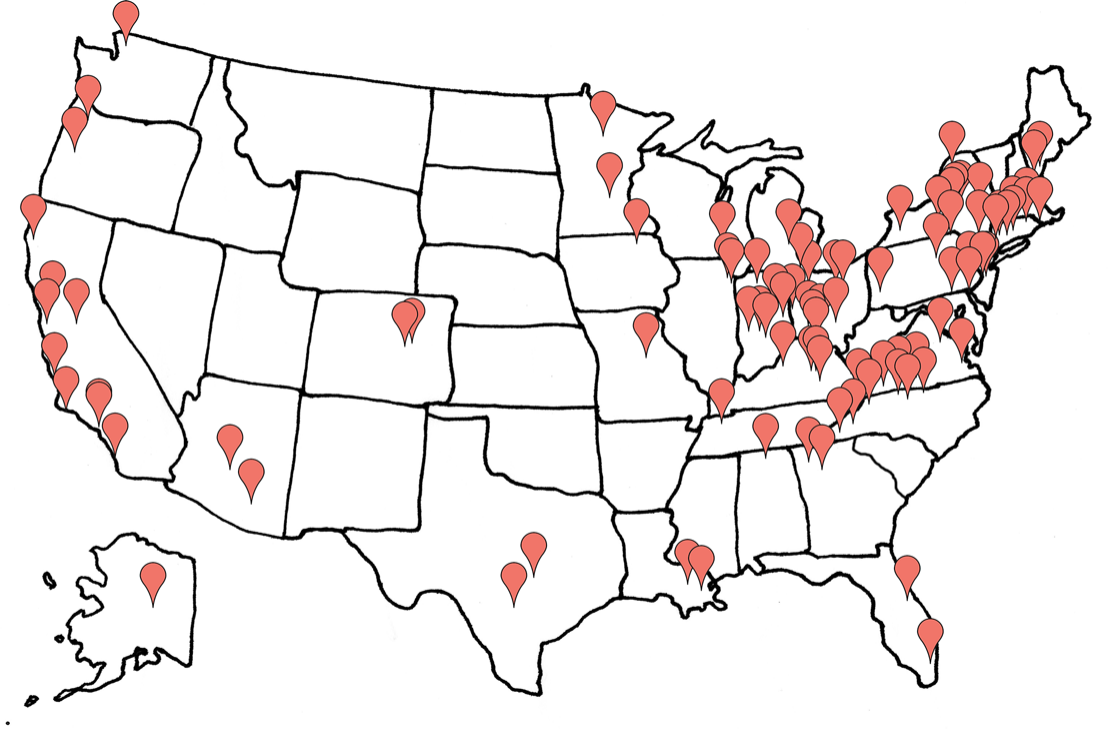
Geographic distribution of schools that participated in the fall 2012 competition.

The fall 2010 and spring 2012 competitions considered in this paper were similar to each other in many respects including broad geographic distribution with particularly strong representation from the northeast U.S. (Fig 1). In both cases, students in participating dorms competed against students in other dorms within each campus to reduce whole dormitory electricity use and in some cases water use. In both years a three-week duration competition with a minimum of once per week meter reading updates was selected because it was felt that this duration was short enough to hold student attention and long enough to allow students to experiment with and learn from conservation strategies in response to weekly (and in some cases real-time) feedback.

The proposal to conduct a national electricity and water use reduction competition among residential student housing was first publicly presented in a conference paper and a workshop at the 8^th^ “Greening of the Campus” conference [8]. The national team that formed to organize the 2010 competition included Lucid, The Alliance to Save Energy (ASE), the National Wildlife Federation (NWF) and the Ohio Board of Regents. Both ASE and NWF had significant experience organizing campaigns on college and university campuses. The Great Lakes Protection Fund (GLPF) provided support for research and pushed to have water as well as electricity included in the competition. The U.S. Green Building Council (USGBC) stepped in to play a significant leadership role in organizing the 2012 competition and subsequent competitions. Lucid is a software company that developed “Building Dashboard”, a web-based technology designed with the goal of making resource use in buildings visible and engaging to a non-technical audience of building occupants (as well as providing analytical tools for building managers).

The CCN competitions were organized by both the national team and local teams within each participating school. In order to ensure that interested schools were adequately prepared and committed to organizing local competitions, the national organizing team required that each school return signed letters of intent to participate from key campus staff, including representatives of the facilities management department, residential education department, student government, and the university administration. Schools were also asked to identify what group(s) within the institution would take principle responsibility for organizing the local events. Although the national organizers provided information and templates for promoting the event, the organizing responsibility was left largely to individual campuses; both effort and approach to organizing differed substantially among campuses. During the months before the competition and throughout the event campus organizers were invited to participate in webinars and conference calls with the national team to plan and discuss implementation strategies on individual campuses. Online instructions for accurately reading different types of utility meters were developed and provided to campus organizers. Manually read meter data was delivered to Lucid and then posted to websites. Researchers at Oberlin College (the authors of this paper) took responsibility for evaluating the efficacy of the CCN program and assessing relationships between behavior conservation strategies and psychological and social metrics.

In both 2010 and 2012 Lucid offered a real-time Building Dashboard as a prize for the campus with the highest percent reduction in electricity. Local campuses determined their own prizes for winning dorms. It would have been logistically challenging to control or to systematically quantify these differences. Anecdotally prizes ranged from bragging rights alone, to ice cream parties for all dorm residents to trophies and t-shirts.

#### 1.6.2. Measuring reduction

Factors influencing total consumption and per capita consumption of a dorm include building size (number of residents and square footage) and the pre-existing magnitude of both discretionary and non-discretionary consumption loads. To control for these factors, percent reduction was used as the standard metric for assessing and comparing performance among dormitories and among campuses. Specifically, to determine reductions, a baseline rate of consumption was established prior to the competition; electricity and water use were monitored during an occupied period shortly before the start of the competition (for example, the two weeks of full occupancy immediately prior to the competition start date). For each participating dormitory, percent reduction at any point during the competition was calculated as the average rate of consumption thus far into the competition relative to the average rate of consumption for that dorm during the baseline period as follows:

Percent reduction during competition=100*(baseline-competition)/baseline

Where “competition” and “baseline” represent the average rates of consumption during these periods (kW for electricity and gallons/hr for water). This number is negative if resource use increases. This approach to quantifying reductions in a dorm effectively controls for: dorm size, preexisting occupant behavior prior to the competition, and for the installed energy consumptive technology in each building. For example, all else being equal, residents of a small dorm might be expected to achieve a similar percent reduction in water and electricity to residents of a larger dorm. Likewise, residents of a dorm with inefficient technologies (e.g. incandescent hallway lighting) will presumably have a higher rate of electricity use during both the baseline and competition than those in a more efficient dorm, but will have roughly similar capacity to reduce consumption through behavioral change. Assuming temperature and natural light do not change substantially between the adjacent baseline and competition periods, this approach also controls for season.

#### 1.6.3. Web interface

In the 2010 and 2012 competitions, the performance of dormitories at each school was displayed and compared on “Building Dashboard”, a publicly available online interface designed and managed by Lucid. Each school received their own Building Dashboard website, which had a unique URL and a “profile page” for each of their participating dorms. Schools could participate for no cost by reading utility meters or extracting data from building management systems and then manually uploading these data to a portal as frequently as they wished (a minimum of once per week was required for participation). Lucid clients have automated metering technology installed such that data are directly transferred to Building Dashboard; clients were able to view dormitory electricity use (and in some cases water) at near real time intervals (between 1–15 minutes after collection). Regardless of whether data were manually or automatically uploaded, each school’s Building Dashboard website allowed participating students to compare patterns of use and reductions in participating dorms on their campus, examine total savings in dorms on their campus, and view national competition standings and statistics.

In addition to allowing comparison of percent resource reduction within and (in the 2010 competition) between all schools, the web interface contained a number of interactive features intended to enhance participation and encourage behavior change. For example, a “commitment widget” introduced shortly after the start of the 2010 competition allowed individual students to select and commit to a range of specific behavioral actions to reduce their environmental impact. Most of these behavioral commitments were directly associated with electricity and water use in dorms (for example “Turn off lights before I leave my room”, “Turn off water while soaping up in shower”). However, some of the commitments were associated with conservation behaviors that would not directly impact water or electricity use (e.g. “Use my bike to navigate campus” and “Recycle my paper and plastic in hallway recycling bins”). The commitment widget was linked to Facebook; if this functionality was enabled by a student then commitments made on a school’s Building Dashboard website also appeared on his or her personal Facebook profile page. Information on the number of commitments made by each student and within each dorm was tracked and displayed. Students could also interact with the website to choose to view patterns of consumption and total savings resulting from the competition in a variety of units including kWh saved, gallons of water conserved, CO_2_ emissions averted, and dollars saved.

#### 1.6.4. Differences between 2010 and 2012 competitions

Although many of the goals and approaches employed to conduct and evaluate the two competitions were similar, there were also important differences that bear on analysis and interpretation. In the 2010 competition, 39 schools with a total of 471 dorms containing approximately 105,000 student occupants participated in the electricity reduction competition (Table 1). In this group, 107 dorms also competed to reduce water. All participating schools agreed to conduct the competition during a common period of November 1 to November 19, 2010. The CCN website and promotional materials framed the 2010 event as representing two distinct levels of competition and one level of collaboration: 1) dormitories within each school competed with each other to achieve the greatest percent reduction in resource use on each campus: 2) schools competed with all other schools to achieve the greatest overall percent reduction in water and electricity use; 3) although no specific CCN-wide goal was set, schools implicitly collaborated to maximize overall savings in electricity and water. A common baseline electricity and water use period from October 15 to October 31, 2010 was generally used. However, schools were allowed to select other baseline periods if a justification could be provided for why a different period was more representative (e.g. a local holiday or other event scheduled during the common baseline rendered this period non-representative). In 2010, the website for each campus provided viewers with the capacity to compare the standing of dorms within each school and also the capacity to compare total reductions among the schools. For schools that manually collected and uploaded data (17 out of the 39 participating schools), the last day of data collection was the closing date of the competition.

**Table 1:** Participation and demographics for 2010 and 2012 competitions.

	2010	2012
Schools completing the competition^a^	39	91
Dorms participating in electricity competition	471 (160)^b^	1,072 (109)
Dorms participating in water competition	107 (30) ^b^	229 (17)
Students living in participating dorms	105,000	197,000
Schools with greater than 10 survey responses	17 (44%)	23 (25%)
Valid survey respondents^c^ (% of total participants^d^)	2,900 (2.5%)	2,600 (1.3%)
Respondents in 1^st^ and 2^nd^ years respectively	57%, 26%	46%, 27%
European heritage^e^	61%	63%
Non-European^f^	17%	25%
Female	73%	68%
Political identification liberal^g^ (and conservative)	45% (26%)	52% (19%)

(a) Schools that initially signed up to participate but did not complete the competition are excluded.

(b) Numbers in parentheses here indicate participating dorms equipped with real-time display technology.

(c) Survey responses were filtered to remove students who did not live in dorms that actually participated in the competition.

(d) The percentage of all students living in participating dorms who completed the survey.

(e) Likely an underestimate as it does not include students who wrote in “white” or “Caucasian”–approximately 9% in the 2012 survey.

(f) Includes respondents self-identifying as African American, Asian, Latino, Native American and Mixed.

(g) Students rated themselves on a 7 point scale from very conservative to very liberal with 4 as neither conservative nor liberal. Percentages reported are for those identified as any degree of liberal (> 4) or conservative (< 4).

The 2012 competition had broader participation than the 2010 event; in 2013, 94 schools competed with a total of 1,072 dorms containing approximately 197,000 student occupants (nearly twice the participating population of the 2010 competition, Table 1). In 2012, 229 dorms also competed to reduce water. Logistical constraints of greater participation precluded concurrent competitions; in contrast to the 2010 competition, in 2012, each school was allowed to select a three week period between February 6th and April 21st to conduct their institution’s competition. Each school was instructed to select a two week occupied period preceding, but as close to the start of their competition as possible, for use as the baseline (avoiding vacation breaks). In 2012, organizers requested that schools also measure rates of consumption during a two week post-competition period immediately following the competition so that the degree to which change during the competition was sustained beyond the competition could be assessed.

As a result of discussions with participants and organizers involved in the first competition and as a result of the constraints of non-concurrent competition periods among schools, the second CCN event was framed as a competition among dorms within schools, but as an entirely collaborative effort at the national level; an overall combined electricity use reduction goal of 1 gigawatt hour of electricity was set for the event (1 gigawatt hour = 10^6^ kilowatt hours). Schools were also provided with the ability to choose to compete against specific peer institutions if the participating schools agreed and coordinated on a common time for the competition. As in the first competition, schools’ Building Dashboard websites provided viewers with the capacity to compare the standing of dorms within each school, and between schools that had agreed to compete with each other. The websites also displayed total reductions and tracked progress towards achieving the 1 gigawatt hour reduction goal. Because of the much smaller number of schools participation in the water competition (229 dorms), no overall goal was set for water use reduction.

### 1.7. Testing for effects of season

Efforts to exclude energy used in HVAC notwithstanding, it is reasonable to speculate that rates of electricity and water use in residential environments as a whole may have a seasonal component. Observed changes in use between a baseline period and a competition period could therefore be affected by changing seasonal patterns of consumption. Potential sources of seasonal dynamics include water used for landscape irrigation and electricity used within dorms for heating, cooling and lighting. A prior assessment by our research group at Oberlin College in Oberlin Ohio (latitude 41°N) indicated no clear seasonal trends in electricity use and therefore concluded that changes observed during these local competitions could justifiably be attributable to behavior change. However, the national competition included schools from a range of latitudes, some of which likely used more electricity for heating and for cooling. It was therefore important that we consider seasonality as a potentially confounding factor in assessing observed change between a baseline period (before the competition) and the competition period itself. The first competition took place during the fall of 2010 and it is possible that certain southern schools shifted from being in a cooling season during the baseline, with electricity used for air conditioning, to a neutral season during the competition period. This could have potentially inflated reductions observed during the competition. On the other hand, many schools in the north shifted from a neutral load to a heating load during this same time period and, if electricity played a major role in heating (as would be the case for electric resistance heating or ground-source or air-to-air heat pumps), then the effects of seasonal change for these schools would have masked behavior induced reductions during the competition. Our analysis allowed us to assess whether such patterns were evident in the absence of a competition.

## Methods of Analysis

### 2.1. Sources of data

Our goal was to collect data that would allow us to achieve the research goals and to answer the research questions described in section 1.5.1. For both the 2010 and 2012 competitions, three separate sources of data were used to assess changes in resource consumption, use of web technology, psychological attributes of participants, and perceived barriers and benefits to conservation:

1. Recorded electricity and water consumption in competing dorms was used to quantify conservation behavior. Data considered included per person consumption in dorms and whole institutions during baseline and competition periods and percent change between these periods. In 2012 we considered consumption during a post-competition period in our analysis for schools that provided this data.
2. Use of the website and website features were quantified by tracking: number of hits to Building Dashboards as a whole, hits to individual dormitories’ pages, duration of visits and use of the “commitment widget”.
3. A post-competition participation survey was used to assess psychological, behavioral and demographic variables. Raffles that included cash prizes were provided as an incentive to participate in the survey.

Practices were exercised to ensure that data collection, analysis and presentation followed ethical standards for research involving human subjects. A research protocol was submitted to, reviewed by and approved by Oberlin College’s Institutional Review Board (IRB). The approved IRB application is available from authors on request. All schools participating in the CCN were informed of the research prior to their commitment to participate. Participation in the CCN was predicated on the public display of resource use data; anyone with web access could view the resource-use data used to assess behavior during the competition. Data were also aggregated over all individuals living in a monitored unit; no data on individuals from either the survey or resource use has been made public in this paper or elsewhere.

Patterns of electricity and water consumption during the fall of 2011 in the subset of dorms equipped with permanent data acquisition and display technology were examined to assess the presence of seasonal effects on resource consumption in dormitories in the absence of competition and to determine whether these effects varied with latitude.

### 2.2. Seasonal effects analysis

The CCN included schools located in diverse latitudes in the continental U.S. (Fig 1) ranging from Louisiana State University (latitude 30.5°N) to University of Alaska, Fairbanks (latitude 64.9°N). In order to consider the potential confounding impact of seasonality we conducted a separate analysis on data for 23 schools during the fall of 2011. This included data for 186 dorms ranging in latitude from 32.2 to 64.9°N. Fall of 2011 lies between the 2010 and 2012 national competitions so we felt this period provided a good measure of underlying seasonal effects that might exist during the competitions. To our knowledge, no resource reduction competitions occurred on these campuses during this 2011 period. Schools included in the seasonal effects analysis are those for which Lucid’s real-time Building Dashboard technology had been installed. With Building Dashboard installed, a continuous hourly record of electricity consumption is stored for each dorm and made publically available on the web. However, only four of the schools considered (with 28 dorms total) included water monitoring. Building Dashboard provided occupants of monitored buildings with access to real-time visual feedback on resource use that could have potentially altered behavior. However, for the analysis we assume that the continuous presence of the technology neither enhanced nor depreciated seasonal patterns; we assume changes in resource use attributable to seasonal effects evident in dorms with real-time feedback are representative of seasonal effects in dorms that do not contain real-time feedback.

For this seasonal analysis we sought to identify a set of common time periods during the fall that were likely to be seasonally distinct from each other. To accomplish this we analyzed freshman orientation and vacation schedules in each of the schools for which we had access to real-time data and were able to identify four periods during the fall of 2011 semester when most of these schools were in session and presumably operating normally. They are designated as follows: “Late Summer” (September 8 to September 22 = 14 d); “Early Fall” (September 22 to October 6 = 15 d); “Late Fall” (November 3 to November 16 = 14 d); and “Early Winter” (November 29 to December 5 = 7 d). The 186 dorms considered in the analysis include only those in which data were available in all four of these periods. In order to assess latitudinal differences in seasonal response, the median latitude of 41.4°N was used to separate what we termed “South dorms” from “North dorms” (93 in each category). Seasonal patterns were separately analyzed for North and South dorms. We used a mixed model analysis of variance to assess seasonal effects and differences between north and south dorms.

For each of the dates listed, rates were averaged from 12:00 am on the start date to midnight on the end date. The periods were constructed to contain approximately the same number of weekend and weekday days so that differences among these periods would not result from differences in resource consumption on weekends and weekdays. Average rates of electricity consumption and water were calculated for each dormitory during each period. While we had sufficient dorms with water monitoring to assess overall effects of season, the small number of schools involved precluded us from assessing latitude effects on seasonal patterns of water use.

### 2.3. Electricity and water use reduction during the competition and sustained reduction following the competition

Cumulative electricity use was measured in kWh and converted to average kW by dividing kWh during each period by time elapsed in hours. This approach provides a direct measure (rather than an estimate) of average consumption rates. Water was measured in gallons and converted to gallons per hour. Results were displayed to competition participants in a variety of other units including dollar savings and CO_2_ emissions averted. The price of electricity per kWh, the price of water per gallon and the carbon emission intensity (lbs of CO_2_ released per kWh) vary regionally. Lucid used regional data for each school obtained from the U.S. Environmental Protection Agency’s “Power Profiler” tool (http://oaspub.epa.gov/powpro/ept_pack.charts) to convert consumption to equivalent CO_2_ emissions that were displayed on the website and otherwise reported.

Reductions were calculated for each individual dormitory in the competition and for the sum of electricity use in all dormitories at each school. Total savings were calculated by multiplying the reduction in rates of water and electricity consumption during the competition relative to the baseline by the duration of the competition. The “grand average percent reduction” was calculated as (baseline–competition)/baseline, where rates used for baseline and competition periods were calculated from the sum of all consumption in all competing dorms in all schools. “Dorm average percent reduction” is the average of the reductions that were separately calculated for each individual dorm. These two approaches produce different statistics because the dorm average percent reduction gives equal weight to small and to large dorms while the grand average effectively weights dorms by the magnitude of consumption and is therefore a more accurate representation of global reductions in a given school.

Because of the nested and dependent structure of the electricity and water use data (school within dorm within time period), statistical analyses used to assess whether reductions were significant were conducted using hierarchical linear modeling (HLM) with the statistical program “HLM” [26]. HLM effectively controlled for variability that occurred among schools and dorms, thus enhancing statistical power. It also controlled for the effect of evaluating multiple variables simultaneously, and allowed for the testing of interactions among variables. Because of the small sample size and large number of potentially confounding variables, water reduction data did not meet the necessary criteria for HLM analysis in 2012. We therefore subjected these data to a paired sample t-test analysis.

In 2012 we requested that campus organizers provide electricity consumption data during a post-competition period so that we could assess the degree to which resource use reductions were sustained beyond the competition. Insufficient data were reported to conduct a similar analysis for water. Prior to analysis we needed to adjust electricity data to account for the reality that different schools reported post-competition data over different time periods. For schools with real-time data, we calculated an average electricity use per person (kW/resident) from hourly data for the 20-day period immediately following the competition. Most dorms did not, however, have real-time data and some reported every five days for a twenty-day period after the competition, others reported every week for two or three weeks after, while others reported once a day for two or three weeks, and still others only followed up for a week or ten days. In these manual-report cases, we computed a post-competition average with the available data that came closest to approximating a twenty day period following the competition.

### 2.4. Use of website

Use of the website was quantified in a number of ways. Questions on the participant survey provided a measure of students’ self-reported use of various features of the website. Tracking metrics incorporated into the website itself provided a direct assessment of website use. Each computer generally reports a unique IP address when it visits a website and this IP address generally lasts for a prolonged period of time (in most cases for the duration of a competition even for “dynamic” IP addresses). The Building Dashboard websites were designed to track visitation data (web “hits”) from each unique IP address and associate it with a particular school and to track visits to the pages for each participating dormitory. For the purposes of analysis we assumed that the majority of hits to a unique dorm web address were attributable to residents of that dorm. Analysis of patters in web-hit data during the 2012 competition are complicated by the reality that different schools participated in the competition during different time periods. For simplicity we therefore emphasize analysis of web use data for before, during and after the 2010 competition. Periods for the 2010 competition were defined as pre-competition (October 15 to October 31), competition (November 1 to November 19) and post-competition (November 20 to December 20). Web use was initially recorded as the total number of page hits, the number of unique page hits (i.e. visits attributable to different IP addresses) and the average duration of page hits. Page hits for each dormitory were expressed on a per-day basis for analysis. In 2012 we considered relationships between frequency of web hits to different dorms and both psychological metrics and behaviors, but did not analyze changes in web hits during different periods. Analysis of variance (ANOVA) was used to assess changes in web use data in response to the competition and correlation analysis was used to assess relationships between web use data and percent reduction in electricity use.

Commitments to specific conservation measures that students made on the website using the “commitments widget” were tracked separately using public tracking mechanisms available through the Facebook interface. In contrast to tracking web hits, students were asked to specifically identify their dorm of residency, so we were able to more definitively link the number and type of commitments made to residents of each dormitory. Correlation analysis was used to assess the relationships between per capita commitments made and resource use reduction in individual dorms.

### 2.5. Participant survey

The participant survey was launched immediately following completion of each competition. Organizers at each school were asked to email a link to all students in dorms that were included in the competition. In the 2010 competition the incentive offered to students for participating in the survey was entry into a raffle in which five $100 prizes were awarded. Because of the larger number of schools participating, the incentive in 2012 was a raffle for twenty $100 prizes. Due to the burden associated with requesting parental consent, only students over 18 years of age were invited to participate in the survey, an approach which excluded young first year students. Several schools choose not to participate in the survey and/or to conduct their own surveys (the results of which are not considered in this paper).

Schools were free to choose whether or not to deploy the participant survey. Students who clicked on the web link for the survey were taken to a consent form that made clear that participation was entirely voluntary and that participants could choose to withdraw from the survey at any point and still be eligible for the reward offered. An affirmative choice statement (effectively written consent) was required on this page in which survey participants acknowledge that they understood the conditions of the survey and were willing participants before they gained access to survey questions. As part of this consent form participants affirmed that they were over 18 years of age. In order to guarantee the anonymity of survey responses, our IRB approved protocol for this survey included a statement to potential participants on the consent page that, “We will only look at your data in aggregate, and will never report individual responses.” In keeping with this commitment, we have made aggregate rather than individual survey responses available.

We were interested in understanding how different groups responded to the competition. The anonymous survey therefore gathered information designed to characterize basic demographic information and political identity. The survey assessed awareness of and experience with the competition, use of different features on the website, conservation behaviors employed during the competition, motivations for participation, resource use consciousness, sense of self-efficacy with respect to conservation, connectedness with nature [19] and perceived barriers and benefits to participation. Specific questions are described in the results section and the complete survey used in 2012 is included as supplementary material (S1 Fig).

Other than the questions designed to solicit basic demographic information, the majority of survey questions were framed as statements and respondents were asked to indicate degree of agreement using a seven point Likert scale (e.g. strongly disagree, disagree, mildly disagree, neither agree nor disagree, mildly agree, agree and strongly agree). The Likert questions, were processed and analyzed in two ways. For reporting, and for chi-square analysis, results were binned into numbers of respondents who expressed some level of agreement or disagreement relative to the total number who expressed an opinion (i.e. those who did not respond “neither agree nor disagree”). For example:

Percent agree=(mildly agree+agree+strongly agree)/(all respondents with an opinion)

We used chi-square analysis to assess whether the number of respondents who agreed with each statement differed from the number of students who disagreed. We also separately calculated an average level of agreement with each statement. This average level is a distinct metric in that it accounts for differences in the strength of agreement. Using the 7-point scale, a neutral level of agreement would have a value of 4.0. Where relevant, we used t-tests to assess whether degree of agreement (i.e. value > 4) or disagreement (i.e. value < 4) was significant. We also used t-tests and ANOVA to assess whether the degree of agreement was greater for responses to certain statements relative to the degree of agreement for other statements. Where appropriate, probability values for statistical tests are reported in parentheses in the results section. Correlation analysis was used to assess relationships between average survey responses for residents of a given dorm and percent resource use reduction in dorms

The questions related to perceived barriers and benefits for conserving water and electricity were open-ended (text responses). Text responses were categorized and coded to identify and quantify themes and patterns. ANOVA was used to compare prevalence of answers that fell into different categories.

Demographic questions were included that relate to gender identity and ethnicity. Participants chose between three possible categories of gender identity: Male, Female and Other. Because the number of respondents answering “Other” to this question was very small (e.g., a total of 6 out of 2,600 respondents in 2012), analysis of differences in gender-based response compared only students identifying in either the male or female category. The question “With what ethnicity do you primarily identify” included seven possible choices: African/African-American, Asian/Asian American, European/European-American, Latina/o, Native American, Mixed Race, Other (please specify). The “Other” category was included to ensure that all possible ethnicity identities could be represented. “Caucasian” and “White” were by far the most common write-in responses in the “Other” category. We did not choose to ask questions about socioeconomic status and therefore made no attempt to control for this factor. ANOVA was used to assess whether there were differences among demographic and political groups in response to various survey questions.

## Results

This section is organized to address the goals and questions outlined in section 1.5 of the introduction.

### 3.1. Electricity and water use (and conservation)

#### 3.1.1. Seasonal effects

**Fig 2:**
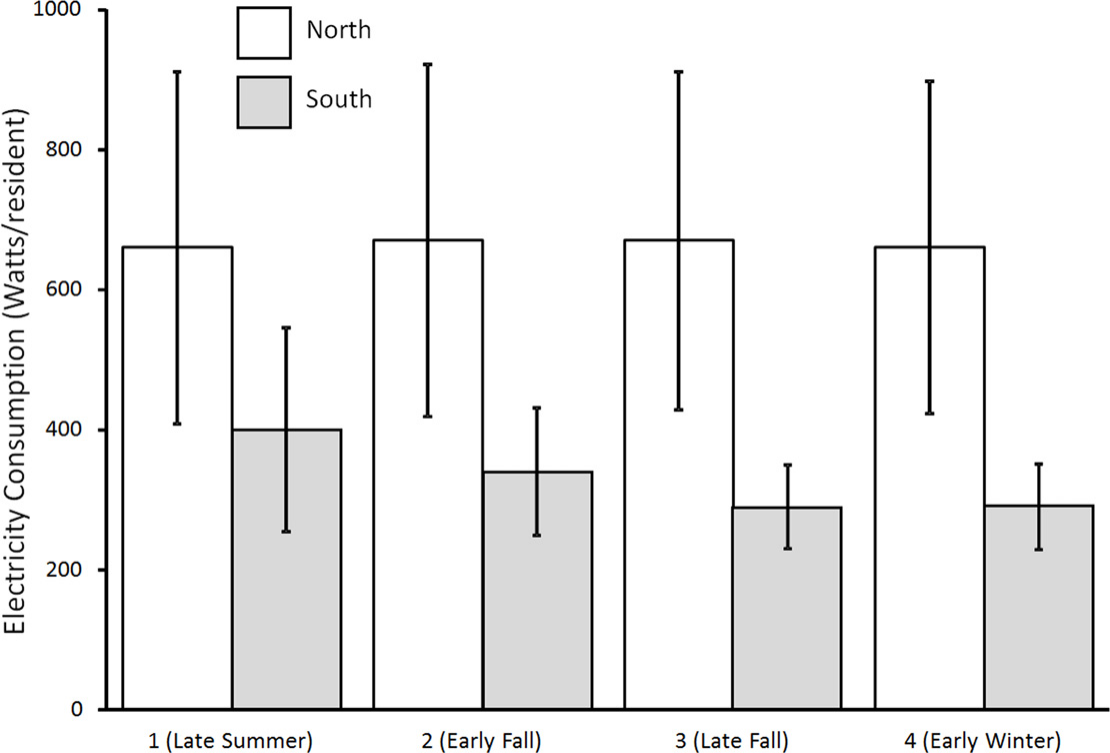
Seasonal per capita patterns in electricity use in dormitories above (“North”) and below (“South”) 41.6°N latitude during the fall of 2011 when there were no competitions. Error bars are 95% confidence intervals.

To evaluate the effects of season independently of competition we first examined the entire set of dormitories included in the fall 2011 (no competition) dataset. We used a 2 (latitude: North vs South) x 4 Season mixed model ANOVA. This model revealed no significant overall effects of season on rates of electricity consumption (p > 0.10). Averaged over all time periods, electricity use in dorms located north of 41.6°N (north dorms) was significantly higher than electricity use in dorms located south of this latitude (Fig 2, F(1, 190) = 5.52, p = 0.02). Although a downward trend in electricity consumption is evident from late summer to late fall in south dorms, this is not statistically significant; no significant seasonal change in electricity consumption between any of the periods was evident in either north or south dorms (p > 0.10 for all possible comparisons of electricity use in different time periods). A repeated measures ANOVA examined differences between season in water consumption, and also failed to find significant differences in rate of water consumption.

While it is possible that seasonal patterns could differ in fall and spring, the reality that no significant seasonal differences were evident in the fall for either electricity or water in either northern or southern dorms suggests that season is not an important predictor of electricity use. It is possible that the non-significant decrease in electricity use during the fall in the south dorms might lead to an overestimate of the effect of the 2010 competition, but the mirrored pattern of increasing electricity use that might presumably be evident in the spring would lead to an under- rather than overestimate of the effects of the competition in 2012. We therefore conclude that significant reductions detected in electricity consumption between baseline and competition periods observed in both 2010 and 2012 competitions (discussed below) are justifiably attributable to the effects of the competition on the behavior of dormitory residents and not to underlying seasonal patterns.

#### 3.1.2. Resource use reductions during the competition

The 2010 competition resulted in total cumulative electricity savings of 510,000 kWh of electricity in the 471 dorms that participated. The grand average electricity reduction was 4.0% while the dorm average was 3.7% (this difference indicating that large dorms reduced by a slightly larger percent than small dorms). Total carbon emissions reductions associated with electricity conservation during the competition was 816,000 lbs. Total electricity utility savings during the competition were approximately $50,000 dollars. There were four dorms that reported unrealistically high increases in water use during the completion (ranging from 70% to 150% increase, with the next highest dorm increasing by only 20%) and these were therefore excluded from analysis. The grand average water reduction for the 103 remaining dorms participating in the water reduction competition was 5.8% while the dorm average was 5.2% (again, large dorms reduced more than small dorms). The competition resulted in total water savings of 570,000 gallons.

In the 2012 competition calculated changes in electricity use for several dorms in which meters were read manually was unrealistically large (for example up to a 320% increase) and we attribute this to human error in meter reading and reporting. In order to be systematic in screening data for meter reading error we assumed that any increases or decreases in electricity consumption during the competition in excess of 50% were a result of error. Using 50% as a filter we excluded 11 outliers leaving a total of 1,084 dorms in the final analysis. We found that the competition resulted in total electricity savings of 1,021 thousand kWh of electricity (slightly greater than the 1 mWh goal set for the competition). The grand average electricity reduction was 3.1% while the dorm average was 3.2%. Total carbon emissions reductions associated with electricity conservation during the competition was 1,622 thousand lbs. Total electricity utility savings during the competition were approximately $100,000 dollars.

As with electricity, we excluded 11 dormitories from water analysis because reported increases or decreases were greater than 50%, leaving a total of 227 dorms for the final analysis. The competition resulted in total water savings of 660,000 gallons. The grand average water reduction was only 0.86% while the dorm average was 2.5% (meaning that small dorms reduced by a much larger percent than large dorms).

It should be noted that savings reported on the official CCN website maintained by Lucid are higher than those reported here because the competition organizers chose to exclude results of dorms that increased electricity or water use during the competition (competition was higher than baseline). Although a case can certainly be made that dorms that increased consumption were obviously not (effectively) participating in the competition, the authors of this paper felt that a statistically valid assessment of the effects of the competition necessitated inclusion of variability below as well as above a zero savings level.

Hierarchical Linear Modeling (HLM) analysis revealed significant reductions in electricity consumption in response to both 2010 (p < 0.01) and 2012 (p < 0.001) competitions. Likewise, HLM revealed significant reductions in water use during the 2010 competition (p < 0.05). As described in section 2.3, because small sample size precluded necessary HLM model assumptions, a paired sample t-test analysis was used to assess water reductions in 2012. These revealed that water use reductions in response to the competition were also significant in 2012 (p < 0.001).

**Fig 3:**
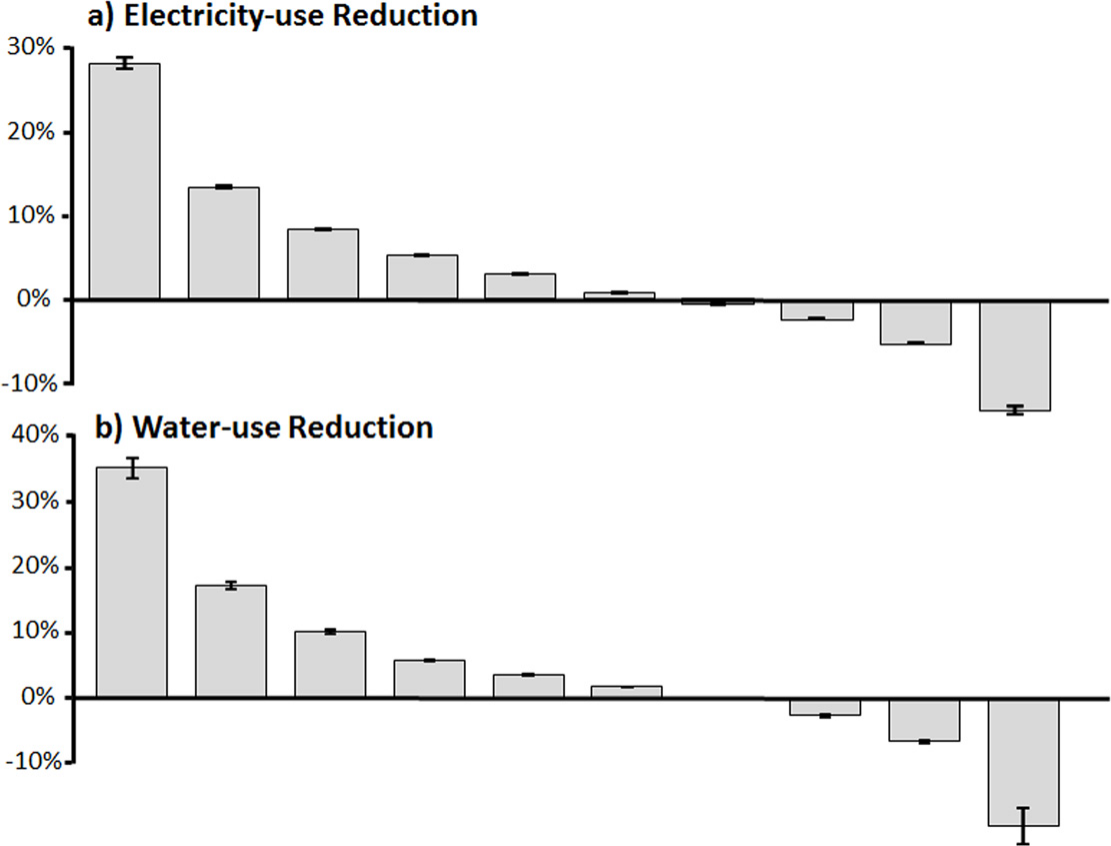
Electricity (a) and water (b) use reductions in the 2010 competition grouped by performance. Each bar is a ten percentile category (47 dorms in each bar for electricity and 10 dorms per bar for water). Error bars are standard error of mean among dorms included in each percentile group.

While significant overall reductions in electricity and water use were evident in response to both competitions, certain groups of dorms performed much better than others. For example when dorms in the 2010 competition are segregated into ten performance levels (Fig 3) it is clear that dorms in the best performing percentile group for electricity conserved more than twice as much electricity in response to the competition as dorms in the next percentile category (28% vs. 13%). Similarly, dorms in the best performing category conserved more than twice as much water as those in the next best performing category (36% vs. 17%). Similar differences in performance categories were evident in the 2012 competition.

#### 3.1.3. Sustained electricity use reductions beyond the competition

HLM analyses revealed that reductions in use of electricity in response to the competition were sustained during the twenty day post competition period; use of electricity in the post competition period was significantly lower than during the baseline period (t (338) = 7.46, p < 0.001).

### 3.2. Direct assessment of website use

**Fig 4:**
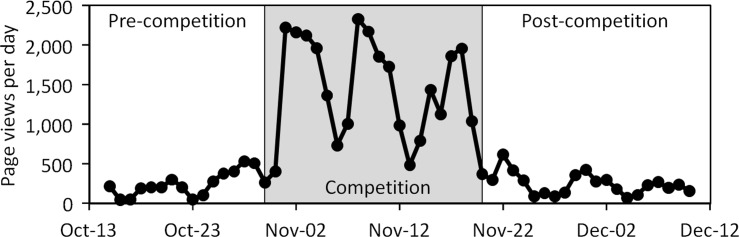
Web traffic to all building dashboards and Campus Conservation National website prior to, during and after the 2010 competition.

In 2010 the total number of visits to the competition website, unique page views to the site (i.e. visits from computers with unique IP addresses) and page viewing time all increased significantly during the competition compared to the baseline and then decreased significantly following the competition (for all web variables, p < 0.01 when extreme outliers were removed, Fig 4). The average duration of a page visit increased from 4.8 to 14.2 minutes between pre-competition and competition periods and then declined to 8.9 following the competition. A total of 550 students made 4,300 commitments to resource conservation on the website during the 2010 competition. Since competitions were held at different times on different campuses in the 2012 competition, it is more difficult to assess global patterns of website use. Nevertheless, as in 2010 significant increases in web views were evident in competition vs. baseline periods in 2012.

### 3.3. Participant survey3.3.1. Basic statistics on competition and survey participation

For both years, survey data were filtered prior to analysis to remove students who did not live in dorms that participated in the competition and students who provided insufficient information (i.e. had not consented to participate or who consented but did not answer any of the survey questions). The fact that 8% of unfiltered respondents in 2010 did not live in competing dorms can be taken as an indication that the incentives for taking the survey were sufficient to entice participation in the absence of any personal interest in the competition. The reality that some school organizing teams did a better job of soliciting survey responses then others is evident in large differences in response rates among schools (Table 1). In 2010 a little less than half the schools had greater than ten survey respondents. In 2010, ten schools accounted for over 90% of survey responses and in 2012, 16 schools accounted for over 90% of survey responses. In 2012 five schools collectively account for 72% of responses. However, in spite of the fact that certain schools are disproportionately represented in the data, for the competitions, the schools that *did* achieve high survey response rates were diverse and included large state schools and small liberal arts colleges. The East and Midwest were well represented in survey responses. Schools on the west coast were poorly represented though several participated in the competition. The size and type of dorm represented by survey respondents was also diverse in both 2010 and 2012. Racial/ethnic diversity was well represented. Females were much better represented than males in survey responses. For both competitions, about half of survey respondents were in their first year of college and about quarter were in their second–perhaps not surprising since underclassman are required to live in dormitories on most participating campuses. Significantly more respondents identified themselves as politically liberal than politically conservative in both competitions (p < 0.001).

Unlike most competitions, students within participating dorms did not individually choose to participate in the CCN; once campus organizers registered a set of dorms within an institution as participants, students living in these dorms were considered competition participants. In spite of the involuntary nature of participation, we found that substantial majority of respondents were aware that their school was participating in the national competition (63% 2010, 68% in 2012) and aware that the dorm in which they lived was participating in the competition (80% 2010, 69% 2012). Survey respondents saw themselves as more aware of the competition then others; respondents estimated that about half of other students in participating dorms were aware of the competition (53% in 2010, 46% in 2012). Respondents estimated that about a third of the students in participating dorms actually changed their behavior as a result of the competition (29% in 2010, 27% in 2012).

#### 3.3.2. Did students visit the website and what features were they most interested in?

A minority of students reported using the Building Dashboard website. In both years, over half of students reported that they had never visited the website (54% in 2010, 55% in 2012). On the other hand, about a fifth of students reported visiting the website more than once per week during the competition (19% in 2010, 20% in 2012). The seven point Likert scale used to assess interest in different features of the web site ranged from “Not interesting at all” to “Very interesting” (Table 2).

**Table 2:** Interest in different features of the website.

	2010	2012
Current competition standing of dorms on my campus ^a^ ^,^ ^b^	92% (34%)	89% (31%)
Current competition standing among schools	81% (18%)	79% (20%)
Graph showing changing patterns of resource use in my dorm	91% (22%)	89% (23%)
Capacity to choose different units for expressing resource use (kWh, CO_2_, $)	81% (18%)	80% (16%)
Feature allowing students to commit to specific actions (linked to Facebook)	78% (13%)	72% (11%)
Competition Tweets made by local and national organizers	61% (10%)	41% (7%)

(a) Numbers reported indicate percentage of students with any level of interest in the feature. Numbers in parentheses are the percent of respondents who indicated the highest level of interest (“very interested”).

(b) Chi-square and t-test analysis indicates that significantly more students expressed interest than non-interest in all features except Tweets. In 2012, both Chi-square and t-test revealed a significantly lower than neutral level of interest in Tweets.

Students judged certain features of the website to be more interesting than others. For example, interest in the display of competition standing among dorms on campus was significantly higher than interest in competition standing among schools (p < 0.01 in both years). Graphs showing changing patterns or resource use in students’ dorms were less interesting then competition standing among dorms but more interesting then competition standing among schools (p < 0.01 for both comparisons for both years).

To assess ease of website use, respondents were asked to assess how easy it was for “others on your campus to find and use” various features of the Building Dashboard website. For all features assessed, significantly more students (p < 0.001 in all cases) felt ease than difficulty in navigating features that included: campus home page (77% in 2010, 70% in 2012), dorm home page (70% in 2010, 66% in 2012), competition results (71% in 2010, 67% in 2012), and commitment to conserve (66% in 2010, 63% in 2012).

#### 3.3.3. Were students motivated and empowered?

In general, students expressed feelings of empowerment, motivation and interest in response to the competition (Table 3).

**Table 3:** Motivation and empowerment to compete and to reduce resource use.

	2010	2012
I felt empowered by the competition^a^ ^,^ ^b^ ^,^ ^c^ ^,^ ^d^	57% (5%)	52% (3%)
I felt motivated to compete	71% (10%)	63% (8%)
Others at my school were motivated to compete	70% (8%)	70% (7%)
The competition motivated me to use less electricity	80% (13%)	77% (11%)
The competition motivated me to use less water^e^	66% (8%)	65% (7%)

(a) Numbers reported indicate the percentage of respondents who express some degree of agreement with the statement while numbers in parentheses are the percent who “strongly agree” with the statement.

(b) Chi-square analysis indicates that significantly more respondents agreed then disagreed with all statements in this table for both years with the exception that feelings of empowerment were not significant in 2012.

(c) In all cases except for the “empowerment” statement, t-test assessment of average levels of agreement reveal significant levels of agreement (p < 0.01).

(d) A t-test revealed that the average level of agreement with “I felt empowered” were not different from a neutral value (p > 0.05 for both years).

(e) This number includes all responses regardless of whether respondents actually competed for water. Only 23% and 22% of dorms actually competed to reduce water use in 2010 and 2012 respectively.

In the literature, perceived self-efficacy was originally defined as the, “strength of people’s convictions in their own effectiveness” in bringing about a target outcome [27]. In our study we operationally measured perceptions of self-efficacy in response to the competition in several ways. One was the degree to which respondents agreed that they felt empowered by the competition. Responses to this first measure–the first statement in Table 3 above–are equivocal. On one hand, in 2010 significantly more students expressed some level of agreement than disagreement with the statement “I felt empowered by the competition”. On the other hand, the degree of empowerment was not different from a neutral value. In contrast, both chi-square and t-test reveal significant agreement with the statement that respondents felt motivated to compete to compete (p < 0.001 both years). When we compared the two, we found that students expressed a significantly higher degree of motivation than empowerment (p < 0.001 in both years).

In addition to the questions about empowerment in response to the competition, two additional questions were employed to operationally assess respondents’ perception of self-efficacy; we asked respondents to rate their degree of agreement with the statements that, “I feel I have relatively little ability to influence [electricity or water] use in my dorm”. In both 2010 and 2012 chi-square analysis and t-tests reveal a significant level of agreement with this statement for both electricity and water (for t-test p < 0.01). However, analysis of variance reveals that in both years respondents felt that they had a stronger ability to influence electricity than water use (p < 0.01). The average of these two measures of perceived self-efficacy were significantly correlated with several other variables assessed in the survey. For example, in both years sense of self-efficacy (measured as degree of *disagreement* with the two survey questions above) was positively correlated with feelings of positivity towards the competition and motivation to conserve both electricity and water (p < 0.001 for all). Self-efficacy was also positively correlated with the number of conservation actions students reported taking during the competition (p < 0.001).

We compared students’ degree of motivation to conserve electricity versus water among respondents who lived in dorms that competed only for electricity and in dorms that competed to reduce both electricity and water use. We found that students were more motivated to conserve electricity than water regardless of whether they were participating in a water reduction competition (p < 0.001 in both years). However, we found that students in dorms competing to reduce both water and electricity were more motivated to conserve water than those who were only competing to reduce electricity (p < 0.001 in 2010 and p = 0.05 in 2012). More interesting, is the finding that in the 2010 competition, students in dorms competing to reduce both water and electricity were significantly more motivated to reduce electricity than students who were only competing to reduce electricity (p < 0.001 in 2010, not significant in 2012).

#### 3.3.4. What factors motivated participation and conservation behavior?

A number of the survey questions were designed to assess the extent to which different factors motivated or discouraged participation in the competition and conservation of resources. For example, the three questions in Table 4 were designed to assess how different types of social comparison influenced interest.

**Table 4:** Influence of social comparison on participation in the competition.

	2010	2012
I was interested in how my dorm was doing relative to other dorms on my campus ^a^ ^,^ ^b^	78% (16%)	76% (15%)
I was interested in how my school was doing relative to other schools	74% (16%)	60% (8%)
The fact that other schools were competing at the same time made me feel like the competition on my campus was part of something larger	73% (16%)	60% (8%)

(a) Numbers reported indicate the percentage of respondents who express some degree of agreement with the statement while numbers in parentheses are the percent who “strongly agree” with the statement.

(b) Chi-square analysis indicates that significantly more respondents agreed than disagreed with all statements in this table for both years. T-test assessment of average levels of agreement reveal significant levels of agreement for all questions as well (p < 0.01).

Although respondents indicated that the competition motivated conservation with respect to all of the questions asked in Table 4, the degree of interest differed for different questions. For example, paired-sample t-test revealed that respondents were significantly more interested in dorm versus dorm comparison on their own campus then they were in the school versus school comparison among campuses (p < 0.001 for both years). In 2010 we asked students what social comparisons might motivate them in future competitions and students indicated some degree of interest in comparison and competition: among individual floors within their dorm (83% agreement), among whole schools in their athletic conference (81%) and among schools otherwise perceived as rivals (88%). Schools perceived as rivals were significantly more motivating than schools in the same athletic conference (p < 0.001).

We found that motivation is related to competition standing. In questions included in the 2010 survey, a significant (p < 0.01) and large majority of students agreed with statements that they would, “more actively participate in the competition if my residence hall was among the top five reducers at my school” (84%) and likewise if their school was among the top five (85%). Non-significant majorities reported that they would be less likely to actively participate if their dorms (53%) or school (52%) were not in the top five.

A set of questions within the survey were used to assess the degree to which different factors motivated competition participants to conserve resources. Students were asked to use a seven point Likert scale to rank their concern for various factors according to the degree of motivation from “not motivating” to “neutral” to “highly motivating”. For both years, we characterized each factor into several non-exclusive categories (Table 5).

**Table 5:** Degree to which different factors motivate conservation.

	2010	2012
My own well being^a^ ^,^ ^b^	88% (21%)	89% (21%)
My health^c^	NA	88% (22%)
The well-being of other students on campus	81% (11%)	84% (13%)
Financial well-being of my College	70% (11%)	71% (14%)
People	88% (18%)	92% (22%)
Animals	87% (24%)	89% (25%)
My country	85% (19%)	88% (22%)
Future generations	91% (33%)	94% (39%)

(a) Numbers in parentheses are the percent of respondents who are “highly motivated” by the factor.

(b) Chi-square analysis indicates that significantly more respondents were motivated than not motivated with all statements in this table for both years. In all cases t-test assessment of average levels of motivation reveal significantly positive levels of motivation for all factors considered (p < 0.01).

(c) The 2010 survey did not include “My health” as a factor.

Analysis of variance revealed that the level of agreement expressed was significantly different for different factors. Concern for future generations was significantly more motivating than any other variable in both years (p < 0.001). Concern for “financial well-being of my college” was significantly less motivating than any other factor considered in both years followed by concern for the well-being of other students on campus (p < 0.001).

The “connectedness to nature scale” [19] is a well-established metric designed to assess the degree of psychological connection an individual feels with the natural world. The version used in the CCN survey consisted of five statements that respondents were asked to rate on a seven point Likert scale from “strongly disagree” to neutral to “Strongly agree”. As examples, two of the statements students were asked to respond to were, “I often feel a strong connection to nature” and “I feel that all living things in this world are connected and I am a part of that”. A high connectedness with nature score is indicative of a stronger psychological connection. We found highly significant (p < 0.001) and positive correlations between connectedness with nature of respondents and the degree to which they expressed feelings that the competition: motivated conservation in general; motivated electricity conservation; motivated water conservation; and empowered conservation actions.

#### 3.3.5. What groups on campus do students believe support conservation?

Students indicated that multiple “groups at my school are trying to decrease energy consumption on campus”. We included fewer categories in 2012, but students expressed a high and similar degree of agreement that all groups considered were trying to reduce electricity use including: faculty (79% agreed in 2010), administration and staff (83% in 2010), graduate students (72% in 2010), undergraduate students (84% in 2010, 88% in 2012), “people living in my dorm” (78% in 2010, 79% in 2012), and “my friends” (80% in 2010, 79% in 2012). Chi-square and t-test analyses both indicate significant level of agreement with the statement that all of these groups were encouraging participants to conserve electricity.

#### 3.3.6. What specific new actions did students take in response to the competition?

Respondents reported taking a variety of new conservation actions during the competition that they “would not have otherwise taken”. The list of action options provided to respondents was expanded from 8 actions in 2010 survey to 26 in 2012. In 2010 the most popular electricity conservation actions taken (and the percentage of respondents who reported taking these actions) were: “turned off lights more often” (66%), “used a powerstrip to completely shut off appliances when not in use” (25%), “talked to hall mates about what they could do to conserve resources” (17%), “got more engaged in environmental activism” (15%), “swapped out an incandescent light bulb for a compact fluorescent one” (10%), and “got rid of a mini-fridge or shared with a neighbor” (5%). The most popular water conservation actions taken in 2010 were: “turned off the faucets when I brushed my teeth” (50%), and “took shorter showers” (37%). Differences in popularity were evident for all actions; turning off lights was a significantly more popular action than using powerstrips, which was more popular than talking to hall mates, etc. (p < 0.001 between all actions listed). Although the set of possible actions was larger and more specific in 2012, the general popularity of different types of responses was similar. For example, in 2012 54% of respondents reported “Used natural daylight whenever possible”, 44% reported “Turned off lights in hallways, bathrooms, and lounges at night”, 40% reported using stairs (instead of elevators) and 13% reported “Talked to hall mates about what they could do to conserve resources”. For water conservation, in 2012 40% reported that they “Turned off faucets when I brushed my teeth”. In 2012 we included actions that were not directly related to performance in the competition and found that students still responded that they engaged in conservation actions that they would not otherwise have taken. For example, 41% reported that they “Recycled appropriate items (plastic, paper, glass, cardboard)” in response to the competition. It is worth noting that in spite of the fact that use of the dashboard website increased significantly during the competition, on the 2012 survey relatively few students reported either visiting the site daily (7%) or encouraging friends to visit the site and make commitments (5%) as actions that they took during the competition. Though less popular than other actions, students did report increased engagement in campus-level initiatives in response to the competition. For example, 8% reported that they “got more involved in student initiatives to reduce campus-wide resource use” and 8% reported that they “learned more about my college's policy on resource conservation”. For both years, we characterized relevant actions as either recurring or one time and as either personal or community. Analysis of variance revealed that in both years recurring actions were significantly more widely adopted then one-time actions (p < 0.001 in both years) and that personal actions were significantly more widely adopted then community actions (p < 0.001 in both years).

#### 3.3.7. How aware are students of resource use and impacts? Do they intend to conserve?

A series of questions were used to assess respondents’ awareness of resource use and intention to conserve. In both years a large and significant majority of students indicated some level of agreement with the statement that “my electricity use has an important impact on the environment”, (86% in 2010 and 87% in 2012, p < 0.001). Strong agreement with the parallel statement for water was likewise evident (86% in 2010 and 88% in 2012, p < 0.001). The degree of agreement with these statements was positively and significantly correlated with a range of other variables including: feelings of empowerment and motivation, feelings of positivity towards the competition, water and electricity use awareness, and connectedness with nature (p < 0.001 for all variables in both years).

Respondents indicated that they are generally aware of their own electricity and water use and make conscious decisions to minimize their use (Table 6).

**Table 6:** Awareness of water and electricity consumption.

	2010	2012
I often think about electricity consumption when I turn a light or appliance on or off. ^a^ ^,^ ^b^	71% (10%)	84% (19%)
I consciously make decisions to minimize my electricity use.	76% (8%)	85% (16%)
I consciously make decisions to minimize other people's electricity use	60% (6%)	68% (10%)
I often think about water consumption when I am taking a shower, flushing the toilet, washing my hands, or using water in other ways.	65% (8%)	72% (14%)
I consciously make decisions to minimize the amount of water I use.	66% (7%)	73% (12%)
I consciously make decisions to minimize the amount of water other people use.	43% (4%)	50% (6%)

(a) Numbers reported indicate the percentage of respondents who express some degree of agreement with the statement while numbers in parentheses are the percent who “strongly agree” with the statement.

(b) Chi-square analysis indicates that significantly more respondents agreed than disagreed with all statements in this table for both years (p < 0.001) except for the final statement. t-test likewise reveals significant levels of agreement for all questions except for the last one (p < 0.01).

The strength of responses to the same questions about water and electricity differed. While there was a significant level of agreement with the statement, “I consciously make decisions to minimize other people’s electricity use” this was not the case for the parallel statement regarding water use. Indeed, in 2010 significant number of respondents disagreed with the statement that they were making decisions to minimize other people’s water use (p < 0.001). Students exhibited a significantly higher level of awareness regarding electricity than water use (p < 0.001). They exhibited a higher level of agreement that they were making decisions to minimize electricity use (their own and others’) than water use (p < 0.001 for both cases, both years). Students in schools that competed to reduce water use were significantly more conscious of water use then in those that competed only to reduce electricity use (p < 0.05 for both years).

In the 2010 survey (but not on the 2012 survey) respondents were asked to assess their intentions to reduce electricity use by expressing their degree of agreement with the statement that “in the next 30 days I definitely plan to use less electricity than I am using now”. Of those who offered an opinion, 81% expressed some degree of agreement with this statement and 12% strongly agreed. A one-sample t-test reveals that the average level of agreement was significantly higher than neutral (p < 0.001).

#### 3.3.8. What did students perceive as the most important barriers and benefits to conserving water and electricity?

The survey included three open-ended questions: “what factors make it difficult for you to reduce your electricity consumption?”, “what factors make it difficult for you to reduce your water consumption?”, and “what do you think are the benefits of reducing your water and electricity consumption?” After responses were reviewed, a classification scheme was developed to categorize each response. Factor analysis was then used as a systematic approach to identify more general clustering of responses into themes. We found that the themes identified as both barriers and benefits of conserving resources during the competition were relatively consistent in 2010 and 2012 (Table 7). Many of the same general issues–the expressed need to consume, lack of personal control (self-efficacy) and apathy (of others but sometimes also of respondents)–were reported by respondents as barriers to both water and electricity conservation. Prominent benefits of conservation that students identified fell into four sometimes overlapping categories: economic, environmental and future generations. In addition to the categories described in Table 7, where possible we also categorized responses to barrier and benefit questions according to the scale–was the issue identified personal, community or global in scale. In both years we found that the perceived barriers to conservation actions tended to occur at the personal and community scale while students tended to perceive the principle benefits of conservation as being global and future-oriented. For example, in 2010 ANOVA revealed that respondents identified benefits at a global scale (10%) significantly more often than at either a community (5%) or personal (5%) scale (p < 0.001). Similarly, in 2012 respondents identified benefits at a global scale (12%) significantly more often than at a community (8%) or personal (5%) scale (all three categories different at p < 0.001).

**Table 7:** Perceived barriers and benefits to conserving water and electricity. Categories were created after reviewing text of survey responses to identify common factors. Percentages are the percent of survey takers who offered responses that fall into each category.

		2010	2012
**Barriers to conserving electricity**			
	“Need” (e.g. to use a computer, lights, etc.)	18%	18%
	Lack of control (over infrastructure, other people, etc.)	11%	17%
	Apathy (community, roommate, personal)	14%	9%
	Convenience (e.g., hard to unplug things, easier to leave things on)	9%	6%
**Barriers to conserving water**			
	“Need” (e.g., to wash hands, clothes, etc.)	6%	8%
	Lack of control (over infrastructure other people)	6%	9%
	Apathy (community, roommate, personal)	7%	3%
	Love of showers (hot showers, long showers)	24%	34%
**Benefits of conserving (both water and electricity)**			
	Economic	20%	25%
	Conservation for its own sake	15%	25%
	Environmental	19%	25%
	Future generations	6%	8%

#### 3.3.9. How did responses differ among different groups of students?

The demographic and political data we collected allowed us to assess whether response to the survey were different for different groups. Several differences were evident between self-identified liberals and conservatives. For example, relative to those who identified as conservative, self-identified liberals exhibited a higher degree of connectedness to nature, expressed significantly stronger beliefs that their electricity use affects the environment, and were more likely to engage in community-level conservation actions (for all of these variables p < 0.01 in both years). However, no significant differences were evident among liberals and conservatives with respect to awareness of resource use, motivation to compete or engagement in individual level actions.

A number of other noteworthy differences were evident when respondents were grouped in various ways. For example, female students were significantly more interested in all of the web features that we asked them to report on then males (p < 0.05 in all cases for both years). First-year students were more motivated to conserve (p < 0.05 in both years) and reported engaging in a larger number of conservation actions than upper-level students (p < 0.005 in both years). Students of non-European ancestry differed from the rest of the population in a number of ways. They were more motivated (p < 0.05 for both years) and generally more empowered (p < 0.01 in 2010; p < 0.1 in 2012) by the competition than those of European ancestry. They were more likely to visit the competition website (p < 0.005 in both years) and more motivated by concern for the financial well-being of their institution (p < 0.05 in both years). In contrast, students of European ancestry were significantly more motivated to conserve as a result of concern for animals (p < 0.05 in both years) and future generations (p < 0.005 in 2012) and made more public commitments (p < 0.01 in 2010; in 2012 commitments could not be traced to individuals, so this test could not be applied).

### 3.4. Relationships between survey responses, web use and measured resource use reductions

We analyzed data to identify relationships between responses to questions posed on the surveys, use of website and reductions in electricity and water use in the dorms in which survey respondents lived. It should be noted that while surveys were assessed at the level of individual respondents, resource-use reductions were measured only at the whole-dorm level. A correlation should therefore be interpreted as being indicative of a relationship between the average response of students in dorms and overall electricity and water use by those dorms. We could identify the unique web hit visitation data of individual computers on individual campuses and we could assess what dorm a visitor was viewing. We could not, however, link individual web hits to individual survey responses. As with survey data, we therefore calculated average web-use data associated with each dorm to identify patterns among variables.

Percent reduction in electricity use in response to the competition was correlated with a broad range of web use and survey data variables. Several of these correlations were statistically significant in one but not both of the competition years. In a few cases, variables were negatively correlated in one competition and positively correlated in the other. Since it is logical to assume that attributes that were significantly and consistently correlated with reductions in both years are indicative of a more robust relationship, our summary below reports only those relationships in which correlation was significant in both years (Table 8). This also minimizes the risk of Type 1 error associated with a large numbers of comparisons. The vast majority of the correlations we report are highly significant, further suggesting the effects are reliable indicators of relationships.

**Table 8:** Factors that were significantly correlated with percent reductions in electricity use in response to the competition in both years. The number of asterisks adjacent to the correlation coefficient (r) indicates significant level: p < 0.001 = ***, p < 0.01 = **, p < 0.05 = *. All significant relationships reported below are positive.

Survey questions and web visitation characteristics found to be positively correlated with electricity reductions in competing dorms	Correlation coefficient r	
Variable	2010	2012
Whether a dorm was competing for water as well as electricity	0.37***	0.21***
Respondent estimate of percentage of students in competing dorms who were aware that the competition was taking place	0.39***	0.36***
Respondent estimate of percentage of students in competing residence halls who altered behavior in response to the competition	0.48***	0.31***
Degree to which respondent felt motivated to participate in competition	0.28***	0.18**
Respondents perception of degree to which other students at their school felt motivated to participate in the competition	0.34***	0.22***
Degree to which respondent was motivated to consume less electricity	0.28***	0.19**
Degree to which respondent was motivated to consume less water	0.16*	0.16*
Degree to which respondents identified global-scale benefits to conserving electricity	0.28***	0.13*
Frequency of self-reported visits to the competition website.	0.27***	0.19**
Degree to which respondents were interested in graphs on the website that showed patterns of resource use in their dorm	0.24**	0.14*
Percentage of students in dorms who made online commitments to conserve resources on the competition website (directly tracked^a^)	0.28**	0.27***
Per capita page views to a dorms’ website during the competition (directly tracked^a^)	0.26***	0.29***

(a) These variables were measured directly by recording and storing web hit data.

We made modifications to the survey questions in 2012 that prevent us from directly comparing correlations for certain variables. For example, in both years we asked students to check a list of conservation actions that they had taken during the competition that they would not have otherwise taken, but this list differed in the two years. In 2010 we found significant correlations between students reporting that they “turned off lights” and percent electricity reduction (r = 0.18, p < 0.05) and significant correlation between students who reported taking shorter showers and percent water reductions (r = 0.15, p = 0.05). Interestingly, although a higher overall percentage of respondents reported that they “turned off the faucet when I brush my teeth” (50% for teeth vs. 37% for shower), turning the faucet off was not significantly correlated with percent water reduction. In the 2012 survey we asked students whether they engaged in a much broader range of more specific conservation actions. And we found that many of these actions were also significantly and positively correlated with reductions in electricity use.

In 2012, the presence of real-time feedback was significantly and positively related to reductions in both electricity (r = 0.10, p < 0.01) and for water use (r = 0.13, p < 0.001). In the 2010 competition water use reduction during the competition was significantly and positively related to the presence of real-time feedback (r = 0.47, p < 0.05). However, electricity use reduction was not significantly correlated with the presence of real-time feedback in dorms in 2010.

## Discussion

### 4.1. Behavior change in response to CCN

#### 4.1.1. Immediate and sustained resource use reductions

A diverse group of schools and a diverse demographic of students participated in the 2010 and 2012 Campus Conservation Nationals and in the post-competition survey (Fig 1, Table 1). Although not a random sample of the total population of college students, this diversity suggests that the observed changes in behavior and in psychological variables are at least broadly representative of what might be observed in colleges willing to participate in a CCN event. Competition results indicate that the objective of enhancing conservation behavior was achieved; students at participating schools significantly reduced electricity and water use in response to the CCN competitions in both years evaluated. A careful assessment of patterns of electricity and water use during non-competition periods indicates that while dorms in northern schools generally consume more electricity than dorms in southern schools, there is no significant seasonal pattern in use of either electricity (Fig 2) or water. This means that observed reductions are attributable to the interventions associated with the competition rather than to seasonal variations in resource consumption. It also means that efforts to weather-normalize electricity data among schools for comparison are likely not warranted.

The grand average reductions in electricity use among all participating dorms– 4% in 2010 and 3% in 2012 – is within the range of what has been observed in response to interventions that employ feedback to minimize residential electricity use [28]. It is important to recognize, however, that the CCN differed in at least four important ways from most prior studies involving feedback. First, in the CCN, feedback was aggregated over whole dormitories instead of individual households. Second, the choice to participate was made at an institutional level; individual dorm residents did not choose to participate and had no capacity to selectively opt out of the CCN competition (other than by simply ignoring the event). Third, students in dorms don’t pay utility bills, eliminating this as a direct financial incentive. Fourth the CCN emphasized competition among dorms, rather than simply the presentation of feedback on resource use as a motivating factor. The first three of these differences might be expected to reduce motivation and result in lower levels of reduction than found in studies of the effect of feedback on households. On the other hand, the emphasis on relatively short-term competition might be expected to enhance reductions, but also to reduce the propensity for sustained conservation following the competition. The fact that significant reductions were observed and sustained beyond the competition period suggests that the approach that CCN organizers took was successful in spite of these challenges.

Significant reductions in electricity and water use observed in response to the competition were paralleled by shifts in self-reported thought and action; survey respondents reported that they were aware of, engaged by, and taking action in response to the competition. For example, a substantial majority of respondents knew that their school was participating in the national competition and knew that the dorm in which they lived was participating in the competition. Likewise, a majority of survey respondents indicated that they had personally taken actions to conserve resources in response to the competition.

The dramatic variability in the percent reductions among different groups of dorms (Fig 3) is one of the more noteworthy results; dorms in the top 10% conserved more than twice as much electricity and water in response to the competition as dorms in the next highest percentile group. The most obvious conclusion to draw is that behavior change can–at least in some circumstances–result in very large reductions in resource use in college dormitories. Why are students in some dorms dramatically more effective than others in curtailing use? Clearly a combination of installed technology and the degree of control that students are afforded set boundaries on what is possible within a given dorm. Nevertheless, the fact that the reductions observed were significantly correlated with survey responses that relate to thought, action and organizing intensity leads us to conclude that the degree of student engagement in the competition as well as other psychological variables are key factors controlling performance. We conclude from the data that efforts to build a stronger level of student engagement in the competition are likely to substantially enhance the resulting conservation.

Whether a response is likely to be sustained over time is an important concern in any intervention, particularly one that employs a short term incentive such as competition. Indeed, prior conservation studies have often observed what has been termed a “rebound” effects, in which consumption returns to pre-intervention levels in response to both installation of energy saving technology (e.g., [29]) and behavioral interventions (e.g., [30]). “Rebound” is also used to refer to return to prior levels after a treatment is removed. It would be reasonable to speculate that the end of the CCN competition might signal a return to pre-competition behavior. We did, in fact, observe a strong rebound of this sort with respect to use of the Building Dashboard websites; a dramatic drop-off is evident following the competition (Fig 4). However, this particular rebound is a logical outcome of the intended use of the website; for the majority of participating schools, the post-competition website no longer presented either useful or new information once the competition ended. So the drop off in use makes sense given that the incentive to compete was removed and comparisons were no longer updated.

But the aim of the competition was to change thinking and to develop habits of behavior related to electricity and water conservation and not to stimulate web traffic. It is therefore gratifying to observe sustained electricity reductions during the two weeks following the 2012 competition; electricity consumption during this period remained significantly below pre-competition levels. Clearly a longer term assessment would be desirable to determine whether or not conservation behaviors decay over time following a competition. But responses to survey questions warrant optimism regarding sustained behavior change; a large and significant number of students expressed the intent to further reduce electricity consumption in the 30 days following the competition. Thus evidence supports the conclusion that the goal of CCN organizers to develop behaviors that extend beyond the competition was realized. It would be valuable for future studies to examine whether shifts in psychological variables also extended beyond the competition.

#### 4.1.2. Reported behavior changes and their relation to observed patterns of resource use reduction in dormitories

A majority of survey respondents reported that, in response to the competition, they took conservation actions that they would not have otherwise taken. It is often concluded that the energy savings potential of one-time measures (such as replacing incandescent desk lights with CFLs) are greater than repeated curtailment behaviors [25]. However, given the tight budgets and transient living situations of college students, it is perhaps not surprising that this population chose to emphasize simple recurring actions, such as turning off lights and turning off water while brushing teeth. In spite of the fact that personal action was significantly more popular than community action, it is noteworthy that certain community actions were widely employed. For example in the 2012 CCN, 44% of students reported that they “Turned off lights in hallways, bathrooms, and lounges at night” in response to the competition. Overall, 17% of students reported talking with fellow dorm members as a strategy that they employed to conserve resources. Although a minority response, it is likewise noteworthy that ten percent of students reported getting more involved in initiatives to reduce campus-wide resource use and nearly ten percent reported learning more about college policy on conservation as a result of the competition. These last two findings indicate that the goal of encouraging greater community engagement was achieved among some participants. This is encouraging, particularly if one operates with the assumption that small groups of organized individuals often have a disproportionate impact on organizational and political processes.

Students’ instincts and choices regarding conservation behavior are sometimes aligned with the impact of these choices and sometimes less so. For example, students’ dominant focus on turning off lights to conserve electricity (by far the most popular action reported) appeared to be well-matched to its impact; the percentage of dorm occupants who reported turning off lights was positively and significantly correlated with percent reductions in electricity use in both competitions. In contrast, we found that there was, in some cases, a mismatch between the most popular actions that students chose to engage in and the impact of those actions. For example, turning off faucets while brushing teeth was a significantly more widely reported water conservation action than taking shorter showers. And yet, the frequency of students in dorms reporting that they took shorter showers was positively and significantly correlated with percent reductions in water consumption while turning off the faucet was not significantly correlated with reductions; taking shorter showers is clearly a far more effective behavior change. This mismatch between popularity of action and impact may be a combined result of misconceptions regarding the relative impact of actions and resistance to taking actions that students perceive as requiring a high degree of personal sacrifice. Indeed, the barrier to shorter showers among college students is apparently great; in their written responses to questions regarding barriers to conservation, many students expressed a very strong affinity (often expressed as “need”) for long showers as a significant obstacle to conservation.

#### 4.1.3. Use of the website and its relation to observed resource use reductions

Building Dashboard websites were designed to function as central organizing tools and to provide a mechanism for providing feedback on how each dorm’s resource use changed over time and compared to that of other dorms. Other studies in which energy use feedback is provided via the web have found a positive correlation between web utilization and electricity conservation [7, 31, 32]. Consistent with this, reductions in electricity use in dorms were significantly correlated with web traffic to the pages displaying data on the performance of these dorms (Table 8). Indirect measures are consistent with this direct measure; a highly significant relationship was observed between self-reported visits to the competition website and electricity reductions in dorms. Likewise, the degree of interest students expressed in data displayed on the website was significantly related to reductions in electricity use.

Use of the interactive component of the website that provided students with the opportunity to make commitment to specific actions was also significantly correlated with electricity use reductions (Table 8). This is consistent with numerous other studies that have found that the combination of goal setting and commitment can enhance residential electricity conservation (e.g., [22, 23, 33, 34]). Anecdotal evidence suggests that students found the particular mechanisms available to make public commitments on the CCN website to be cumbersome. Furthermore, a number of students expressed negative views towards the required use of Facebook for making commitments. We believe that there is considerable opportunity for enhancing user experience and utilization of public commitment within future resource use reduction competitions.

Students expressed a high degree of interest in most of the features displayed on the website (Table 2), but particularly in visual depictions of how their dorm was performing relative to other dorms on their campus and in the pattern of change over time in their dorm’s resource use as depicted in time series graphs. Although the design of this research did not allow for a direct measure of effects of other specific features of the website, other controlled studies have found that the depiction of patterns of use over time (a feature of the Building Dashboard display) and incentives (not included in the display) result in significant reductions in electricity use [31].

Given the significant correlations between utilization of web features and resource conservation it is particularly noteworthy that a minority of students made regular use of the website; only 19% of students indicated that they visited the Building Dashboard website more than once per week during the competition. What explains the expressed interest and strong relationship between web use and observed reductions in spite of low overall levels of regular utilization? In addressing this question it is important to recognize that use of the website has the potential to serve as both cause and effect of engagement and empowerment. Presumably students who were motivated by the competition were more interested in the website than students who were not and in this sense it is possible that the correlation is an effect of successful competition rather than a cause of it. From this perspective dorms that were particularly engaged were likely to have students who visited the website. On the other hand, if we assume that the website provided valuable information that enhanced the desire and ability of students to successfully conserve then it may be the case that only limited contact is necessary for a dorm community to garner this information. For example, it is plausible to envision a situation in which particularly eager students glean information from the website then share this information among fellow dorm residents via other information conduits such as direct conversation, email and text messaging. This makes sense in the context of a competition in which new resource use information for most campuses was only posted once per week. Other researchers have found that peer networks can be an important component of successful conservation efforts (e.g., [24]).

It appears that the presence of more frequently updated information does, in fact, enhance resource use reduction. In both competitions water use reduction was significantly and positively correlated with the presence of real-time (vs. weekly) feedback. We also observed a significant correlation between electricity use reduction in dorms and the presence of real-time feedback in 2012 (results were equivocal in 2010). The general observation that a finer temporal resolution of feedback leads to larger reductions is consistent with the findings of a number of other studies (e.g., [30]). Improvements in technology and reductions in the cost of monitoring are likely to make it easier for additional schools to add this feature. Certainly the availability of high resolution continuous data would substantially enhance the ability to assess the impact of behavioral interventions as well as enhancing the capacity to assess the impact of energy efficiency technologies.

### 4.2. Psychological factors associated with and responsible for resource use reduction

The findings discussed above indicate that the core programmatic goal of using the CCN event to induce electricity and water conservation through behavior change was achieved and that use of the website and real-time feedback enhance behavior change. It has often been the case that the effectiveness of resource conservation programs is assessed with no attempt to evaluate underlying psychological determinants [30]. A key additional goal of this study was therefore to better understand the psychological factors that contribute to or are associated with conservation behavior. A wide range of behavioral models can and have been used to explain factors responsible for behavior change (for a review related to feedback interventions see [18]). As discussed in section 1.2, we assumed four linked but distinct factors would be necessary to achieve resource conservation behavior in the CCN. Specifically we assumed that a successful program would: engage (catch attention of the target audience), educate (communicate information on what, why and how behavior should change), motivate (enhance desire to change behavior) and empower (increase perception and reality of self-efficacy and suggest concrete and actionable behavior).

#### 4.2.1. Engagement, education, motivation and empowerment

Several different survey questions are relevant to addressing the question of how these psychological variables are related to competition, how they relate to conservation behaviors and how they relate to each other and to additional relevant variables. In this section we examine the effect of the competition on each of these variables and how these variables relate to performance in the competition. In the subsequent section we examine relationship among variables.

*Engagement*: Measurements relevant to assessing the degree of engagement include web hit data and several survey questions in which students expressed their own and their perceptions of other students’ degree of knowledge, awareness of and interest in the competition. Following the competitions, students expressed a high degree of awareness and interest in resource consumption and in conserving resources (Table 6). For example, survey respondents reported that they were highly aware of environmental impacts of resource consumption; over 85% agreed with the statement that their personal consumption of electricity and water have important impacts on the environment. Students also think about consumption when they use these resources and large and significant majorities report making conscious decisions to minimize their own and other people’s electricity use. We take the high level of interest as a sign that college dorms are fertile ground for engagement in resource reduction competitions. All factors we measured indicated that students were significantly engaged by the competition (e.g., Tables 2 and 4). Furthermore, a variety of measures of student engagement in the competition were positively and significantly correlated with electricity reduction during the competition (Table 8) including awareness that the competition was taking place and actual and self-reported use of and interest in the website.

*Education*: Because different campuses designed very different educational programs around their local CCN events, we did not feel that we could independently measure relationships between competition and education. However, in a previous study at Oberlin College we focused more squarely on the impact of resource reduction competitions on knowledge of resource use and on self-education related to conservation behaviors. In that study we found that competition had no measurable effect on people’s factual knowledge of resource use and its environmental impact but that a significant majority of participants reported that they had taught themselves new conservation behaviors during the competition that they intended to continue employing after the competition ended [7]. Self-reported intent to continue conservation behaviors learned during the CCN competitions is consistent with these prior findings.

*Motivation*: In general we observed that participants expressed a high and significant degree of agreement with statements that they and other students on their campus were motivated by the competition to engage in additional conservation measures (Table 3). At the same time, a deficiency in motivation was also identified by many students as an obstacle; in open-ended questions respondents indicated that apathy (i.e. lack of motivation) of dorm occupants, roommates and sometimes of the respondents themselves were major barriers to conservation (Table 7). In spite of this apparent challenge, as with engagement, both the self-motivation and the perceived motivation of others within dorms were significantly correlated with actual electricity reductions of the dorms (Table 8). This was true both for motivation to participate in the competition itself and motivation to consume less electricity and less water. Interestingly, perceived benefits of conservation were skewed towards global and future concerns while perceived barriers tended to be local (Table 5 and Table 7). The fact that different campuses offered different prizes and rewards for winning dorms might be seen as a potentially confounding factor in considering motivation. However, in our open-ended survey question regarding the benefits of reducing resource consumption during the competition, so few students mentioned prizes that this was not identified as a category worth separating in our analysis; external prizes did not appear to be an important motivating factor for students.

*Empowerment*: Responses to questions designed to assess perceptions of self-efficacy–strength of people’s convictions in their own effectiveness in bringing about the target outcome of resource use reduction–are more equivocal than are responses to questions related to engagement and motivation. On the one hand, more respondents expressed some level of agreement than disagreement with the statement that the competition was empowering (Table 3). On the other hand, many students strongly disagreed with this statement. Indeed, when measured as average strength of agreement we found that the strength of disagreement was stronger than the strength of agreement with this statement. Furthermore, there was also significant agreement with the statement, “I had limited ability to influence [resource] use”. Finally, in open-ended questions, lack of perceived control over dormitory infrastructure and over other people was a commonly identified barrier to reducing electricity and water use during the competition (Table 7). In contrast to engagement and motivation, measures of empowerment were not consistently correlated with electricity reduction.

As noted previously, CCN differed from many other residential feedback interventions in that resource use was aggregated over large groups of dormitory residents rather than over households and this raises interesting questions about the potential importance of individual self-efficacy (belief that the individual can achieve a goal) and collective efficacy (the belief that groups that the individual belongs to can successfully accomplish a goal). Although data from our study supports the view that finer scale feedback enhances motivation, our findings that whole-dorm feedback can motivate conservation is consistent with other research findings that feedback on the performance of larger groups can effectively motivate behavior change [35]. The important distinction between disaggregated and group-level feedback is that group-level feedback triggers a lower expectation that changes in individual behavior will necessarily be evident in the feedback received. On the other hand, group-level feedback may promote a sense of collective outcome expectancy, or the expectation that the collective actions of group members may be successful in bringing about a desirable result [36].

Behaviors that students reported engaging in during the competition support the complementary importance of both individual and collective efficacy. On one hand, individual-level behavior changes (such as turning off water while brushing teeth) were the most commonly reported, indicating a focus on self-efficacy. On the other hand, significant fractions of students reported taking community-level actions that they would not otherwise have engaged in as a result of the competition, indicating a strong belief in collective efficacy. Some of these responses were highly local (e.g. the 15% who “talked to hall mates about what they could do to conserve resources”), but some were broader in scope (e.g. the 15% who “got more engaged in environmental activism”). While we have no mechanism for assessing the extent to which community-level behaviors might have been sustained beyond the CCN event, we are optimistic that the experience of this broader scale of action may set presidents for continued future engagement at this larger scale.

#### 4.2.2. Relationships between motivation and empowerment

Relationships evident among engagement, motivation and empowerment and between these and other variables imply interesting psychological dynamics at play in response to the competition. These relationships also point towards strategies that might be employed to enhance impact in future competitions. For example, we found that awareness of the environmental impact of resource use was significantly correlated with both empowerment and motivation to participate in the competition. Although we cannot be certain of the nature of causality implied by this correlation, results at least suggest that education about resource use impacts may be a valuable approach to enhancing self-efficacy.

Of particular interest are differences in the relative strength of motivation and empowerment and relationships between these measures. A positive perception of self-efficacy is generally thought to be a necessary condition for behavior change [13]. Although our study was not designed to assess the extent to which elevated feelings of self-efficacy stimulate motivation and action we did find that measures of empowerment were positively and significantly correlated with motivation and with the number of new conservation actions that students reported engaging in.

The reality that students expressed significantly higher levels of motivation than empowerment taken together with other survey responses provides a clear indication that many students were frustrated by what they perceived to be their limited capacity to affect meaningful resource use reductions through behavioral choices available to them in dorms. This suggests a conundrum in the use of sociotechnical feedback as a tool for promoting conservation. Program designers who employ feedback typically assume (often implicitly) that introduced feedback increases both motivation and self-efficacy. The specific assumptions are that enhanced awareness of current levels and patterns of resource consumption will motivate a desire to conserve while enhanced ability to directly observe cause and effect relationships will minimize the discrepancy between perceptions of self-efficacy (how much a person believes that they can take actions to achieve a goal) and actual efficacy (the degree to which actions taken by the individual actually achieves this goal). There are two potential problems with these assumptions related to self-efficacy. The first is that recipients of feedback may not have sufficient knowledge about mechanisms responsible for resource consumption to accurately interpret information embodied in feedback. The second, and potentially more vexing potential problem with feedback, is that the recipients may learn that their actual ability to affect change is more limited than they had previously understood and this new knowledge may thereby actually *reduce* rather than enhance their perceived self-efficacy. We believe that both of these may be operating in student’s response to the competition. For example, survey respondents expressed both a lack of clarity regarding what actions might be most effective at reducing resource use and difficulty in seeing the effects of individual or even collective action in the feedback provided on the standing of their dorm during the competition.

A danger is that increased motivation to conserve combined with decreased sense of self-efficacy may lead to frustration. Ultimately, alleviating this frustration requires some combination of providing students with greater control and providing clearer information about decisions that they can make that will meaningfully reduce consumption. As an example of enhancing control, at Oberlin College we have found that providing a manual override switches that allow student to turn lights off in rooms with occupancy controlled lighting leads to greater student satisfaction (and presumably reduced electricity use). An effective promotion of competitions might also benefit from focusing on building a stronger sense of collective as well as individual efficacy. This would be consistent with our finding that student engagement in community-level conservation actions that they would not otherwise have engaged in was positively correlated with reductions in electricity use (Table 8).

A final issue related to the impact of self-efficacy and motivation on conservation behaviors emerging from this study relates to perceptions of need. Even if an individual has both motivation and a perceived ability to act in ways that would conserve resources, this may conflict with perceptions of need. For example, we found that the most common barrier students identified to conservation for both water and electricity was a perception of the fundamental need to consume these resources. In open-ended responses students identified a need to use electricity and water to accomplish basic necessities such as using a computer for studying and for lights, using water to wash hands, body and clothes. Enhancing conservation is clearly contingent on changing actual needs (e.g. by providing better natural lighting so that electrical lighting is less necessary) as well as on changing perceptions of what is needed (e.g. reducing perception that long showers are a fundamental need).

#### 4.2.3. Differences in the response based on gender, political identity and ethnicity

Perhaps not surprisingly different groups of people think differently about resource use, and experience different degrees of engagement, motivation and empowerment in response to conservation initiatives. Our survey finding that women report being significantly more aware of their resource use than men is consistent with the work of others who have found that women tend to identify a stronger degree of concern for the environment than men [37]. Likewise, it is interesting, though perhaps not surprising that in a number of ways self-identified liberals and conservatives think and act differently with regards to the environment and conservation; politically liberal students have a stronger degree of connectedness to nature, are more concerned with the environmental impacts of resource use and have lower levels of baseline electricity consumption. Of equal interest are the ways in which liberal and conservative students do *not* differ; we detected no differences in awareness of resource use or in their degree of motivation to participate in the competition. This suggests that competition may be an effective means of engaging students across the political spectrum.

Demographic patterns related to ethnicity and race are also interesting and suggestive. For example, the finding that students of non-European ancestry were generally more motivated, empowered and likely to visit the competition website than those of European ancestry warrants further examination. Differences in expressed motivations are also important. For example, those of European ancestry were significantly more motivated by concern for future generations and animals than those of non-European ancestry. In contrast, those of non-European ancestry were significantly more motivated by concern for the financial well-being of their institutions than those of European ancestry. We did not gather data that would have allowed us to control for economic backgrounds, but it is at least possible that some of the observed differences are partially explained by differences in affluence among these groups. In any case, observed differences suggest diverse messages and diverse strategies are important to effectively engage and motivate the full diversity students present at participating institutions.

#### 4.2.4. Social norms, scale and connectedness with nature as factors motivating participation

Survey responses indicate that social norms–perceptions and beliefs about how members of a group think and behave relative to others–were a significant determinant of motivation to participate in the competition. This is evident in the high percentage of survey respondents who indicated that that they were interested in how their dorm was performing relative to other dorms and how their school was performing relative to other schools (Table 2). The highest and most significant correlation coefficients we observed between survey responses and electricity reductions are all measures of social norms (Table 8)–for example electricity reduction is correlated with survey respondents’ estimates of whether other students had altered behavior in response to the competition. Consistent with the findings of other researchers (e.g., [10]), we found that normative variables were significantly more motivational than financial considerations. The fact that financial considerations were not a very important driver of behavior is actually consistent with findings of studies conducted in residential environments in which participants do, in fact, pay a utility bill [9]. Interestingly, it is clear from survey responses that resource conservation is already a broadly held social norm on campus. Student responses indicate significant levels of agreement with the statement that different groups on campus–including faculty, administration, staff and other students–are all trying to decrease electricity use on campus.

Survey responses suggest that the scale and physical and psychological closeness of relations between self and comparative groups were important determinants of motivation. For example, students were significantly more interested in dorm vs. dorm comparisons on their own campus than in school versus school comparisons. They also expressed a high degree of interest in adding even finer scale comparison by including floor versus floor comparison. In terms of perceived closeness of relationships, students were significantly more interested in comparing their school to institutions that they perceived as direct rivals rather than to schools that were simply in the same athletic conference. This finding that the propensity to respond to normative feedback is enhanced as the norm becomes more contextually specific and local to the individual under consideration is consistent with the findings of other researchers (e.g., [21, 24]).

An important additional normative relationship we observed is that motivation to participate was related to the standing of respondents’ dorms and schools in the competition; significant majorities of students indicated that they would be more motivated to participate if their own dorm or school were among the top five performers. This finding suggests the value of constructing competitions in a way that results in multiple winners in different categories and minimizes the number of dorms and schools that perceive themselves as losers. The finding also validates the operational decision to shift from a competitive to a collaborative framing of the between-schools component of the competition in the ‘12 vs. ‘10 competition. Norms, perceptions of self-efficacy and the scale of competition are related in ways that are potentially complex and need to be carefully considered. For example, in order to minimize frustration, it may be advantageous to structure resource use reduction events as competitive in situations in which self-efficacy and collective efficacy are high and collaborative in situations in which perceptions of efficacy are low.

Survey questions designed to identify the barriers and benefits to resource conservation also revealed interesting scaling patterns; issues that students identified as motivating conservation were skewed towards global and future concerns (rather than local and immediate) while barriers to conservation that students identified tended to be at the personal and community scale. For example, in response to agree/disagree statements, concern for future generations was significantly more motivating to students than any of the other factors considered (Table 5), while concern over the well-being of other students on campus was among the least motivating. Indeed, concern over global-scale benefits of electricity conservation was the only motivating factor that students identified that was significantly correlated with electricity use reduction.

Students’ degree of emotional connection with nature [19] was associated with many other factors. For example, we found highly significant and positive correlations between connectedness with nature of respondents and the degree to which they expressed feelings that the competition: motivated conservation in general; motivated electricity conservation; motivated water conservation; and empowered conservation actions. Other research conducted by our group has demonstrated that the degree of psychological connection with nature is, in some cases, negatively correlated with baseline levels of electricity consumption and can, in some circumstances, be associated with lower levels of response to competition. An explanation is that those who have a high degree of intrinsic motivation to conserve may either already be exhibiting conservation behaviors or may simply not be motivated by the extrinsic factors associated with competition. In any event, further research is warranted to assess the degree to which connectedness with nature might influence performance in competitions.

#### 4.2.5. Differences in response to water and electricity competition and spillover effects leading to conservation of other resources

Differences in students’ thoughts and actions related to water and electricity may reflect differences in perception of the importance of these resources and the way the competition itself was promoted. In general we found that students were significantly more motivated to conserve electricity then water, even in dorms that competed to reduce both resources. Consistent with this pattern in motivation, student awareness of, and effort to minimize consumption was significantly greater for electricity then for water (Table 6). It may well be the case that the enhanced level of interest and motivation to conserve electricity reflects the perception that electricity consumption is more damaging to the environment. Certainly this is evident in the significant correlation between electricity use reduction during the competition and perceived global scale benefits of such action (Table 8). But it is also important to acknowledge that national and also local promotion for the CCN events placed greater emphasis on conserving electricity and so the framing of competition itself may have biased students’ perceptions of the relative importance of conserving these two resources.

The fact that respondents felt that they had a stronger ability to control electricity than water use in their dorms and a stronger ability to control community electricity use than to control community water use has potentially important implications for programs targeting behavior change. For the most part these contrasting perceptions of self-efficacy appear to align with reality, particularly as they related to influencing the behavior of others and the behavior of groups. While an individual student can make personal choices to conserve both water and electricity, the opportunity to influence others is different. For example, many students reported turning off lights in public spaces to conserve electricity, but there is no clear parallel for this with respect to water use. Open-ended survey responses suggest that students are more inclined to encourage others to engage in personal conservation actions associated with electricity (for example using task lighting or power strips) than encouraging personal actions associated with water (such as shorter shower or different use of toilets). Suggestions related to water are likely perceived as a more personal and intrusive. This distinction in attitude towards resource use is reflected in differences in students’ stated effort to influence other people’s choices. While a significant majority of students agreed with the statement that they consciously made decisions to minimize other people’s electricity use, a significant majority of students *disagreed* with a parallel statement about minimizing other people’s water use (Table 6). These findings suggest that different strategies of messaging, with different levels of appeal to self- and collective-efficacy, are appropriate for encouraging conservation of these different resources.

The discussion above indicates that students think and act differently in response to different resources. An important related question to consider is, does a focus on conserving one resource affect conservation of other resources? Survey responses suggest that both spillover effects and positive synergy did in fact occur. In terms of spillover, students indicated that they were motivated to exhibit new behaviors that extended beyond those that they were directly rewarded for. For example, when asked what new conservation activities they engaged in as responses to electricity and/or water use reduction competitions survey respondents reported engaging in behaviors that have no clear relationship with electricity or water used in dorms including increased bicycle use and minimization of food waste. A full 41% of survey respondents reported that they increased recycling paper and plastic in hallway bins as a behavior change that they employed in response to the electricity and/or water use reduction competition–an action that clearly has no relationship to performance in the competition. In terms of positive synergy, we found that dorms that were configured by organizers to compete to reduce water as well as electricity conserved significantly more electricity than dorms set up only to compete for electricity reductions.

The degree to which this kind of conservation spillover from one target resource to another occurs is likely partially determined by how a conservation event is promoted. While the CCN focused on electricity and to a lesser extent water conservation, the overall framing of the competition centered on environmental benefits of conservation. And, as discussed earlier, many participating schools found value in integrating CCN into broader awareness programming that combined a variety of events related to environmental sustainability. This makes it challenging to definitively separate spillover effects of the electricity and water use reduction competitions from response to other pro-environmental interventions that took place on some campuses. Nevertheless, although we have no way to assess how the various combinations of promotion influenced reductions, others have found that combined interventions often lead to greater conservation because different individuals experience different barriers and are motivated by different factors that may be addressed by different interventions [22, 25].

## Conclusions

Facilitating the transition to more sustainable patterns of resource consumption is one of the principle challenges faced by this generation of college students. An effective response requires change in both thought and behavior. Programs like the CCN provide one of many approaches to eliciting such change. Fortunately, an increasing body of theory and research exists that can be leveraged to make interventions successful. Indeed, a number of recent publications focus explicitly on how knowledge from social psychology and communications can be used to improve program design (e.g., [20, 38, 39–41]). With careful planning, programs such as the CCN can be designed to test and advance as well as to apply relevant theory.

Motivating behavior change that reduces resource use on campus is important in part because of the direct benefits to the environment such as reductions in greenhouse gas emissions and other pollutants. But the larger importance of conservation programming may be in encouraging habits of pro-environmental thought and action in students that extend beyond college years and into a variety of other contexts. While these larger scale and longer term impacts are difficult to quantify, the results evaluated here provide promising evidence that initiatives such as CCN can, indeed, elicit changes that extend beyond electricity and water conservation and beyond the competition period. While certain of the findings reported here may be restricted to the unique environment of college campuses, a number of the findings may extend to adults in other residential settings. For example, as we point out in the discussion above, our findings that relate to self-efficacy, collective-efficacy and social norms are consistent with and enhance findings from many other studies conducted in non-college settings.

A key feature of the CCN program was the introduction of feedback–information that allowed participants to track the results of their conservation efforts and then further adjust their behavior in response. The renowned engineer W. Edward Deming has been widely (and incorrectly) credited with the statement that, “If you don’t measure it, you can’t manage it”. The results of CCN are consistent with a growing number of studies that demonstrate that monitoring and displaying resource consumption in the residential environment can lead to significant reductions in resource use (e.g., [28]). However, we are struck by the fact that students in the best performing 10% of dorms reduced both water and electricity consumption by greater than twice that of the next best 10%. This highlights the reality that responses to conservation programs that incorporate feedback are highly variable. While we hope that this paper sheds some light on factors associated with success in the best performing groups, further research is clearly necessary to advance our understanding of the components of feedback and competition that maximize resourced use reductions and enhance desirable spillover effects into other contexts.

## Supporting Information

S1 Fig 2012 Survey Form for Campus Conservation Nationals. (PDF)
