# Supplementary material for: Electricity and Water Conservation on College and University Campuses in Response to National Competitions among Dormitories: Quantifying Relationships between Behavior, Conservation Strategies and Psychological Metrics
Source: PLoS One. 2015 Dec 16;10(12):e0144070. doi: 10.1371/journal.pone.0144070 (PMC4687655; doi:10.1371/journal.pone.0144070)
Supplement: S1 Fig — (PDF) [file pone.0144070.s001.pdf]

campus conservation nationals 2012

Consent Form

Welcome, and thank you for your interest in contributing to our research. We are evaluating the effectiveness of the Campus Conservation Nationals resource reduction competition that your school just participated in. We will be looking to see what kinds of student characteristics are associated with resource reduction, and what parts of the competition were most compelling to students. This online study will take no more than 10 minutes to complete. You will be asked to answer some questions about yourself, your resource use, your campus, and your views of the competition. To thank you for your time, we will enter your name in a drawing to win one of 20 $100 prizes.

There is no risk of harm or discomfort in this study. One benefit of participating is that you can feel good about helping us improve future competitions.

You are free to quit at any time during the study and still be entered in the drawing. Your answers will be completely confidential, and will only be used for research purposes. We will only look at your data in aggregate, and will never report individual responses. We will provide your campus competition coordinator with data that we collect from people at your school, but all identifying information will be removed first. There will be no way for them to trace your responses to you.

Please note: You must be 18 years of age or over to participate in this study.

If you should have any questions about this study, please contact **Principle**

Investigator Dr. Cindy Frantz (440 775 8499, cindy.frantz@oberlin.edu), or Heather Hogan (Associate Dean of the College of Arts and Sciences and **Chair of the Institutional Review Board**, 440 775 8410, heather.hogan@oberlin.edu).

- ○ Yes, I consent
- ○ I do not consent

If you would like to be entered into the drawing for one of the $100 cash prizes, please enter your email address below. Your email address will only be used to award the prizes, and will not be stored with your data.

[ ]

The Building Dashboard gave you the opportunity to log into the competition website using your facebook account. This means that posts you made through the dashboard could be traceable to your email address. Would you be willing to have this information paired with your survey responses via your email address? Allowing us to pair your data is optional but helps us to assess participation in various aspects of the competition. Even if you choose not to allow us to pair your public posts, you are still eligible for the cash prize, and your survey data is still useful to us. Once again, we will not share your email with anyone, your responses will be rendered anonymous and we will delete your email from the data file once we have matched data and awarded prizes.

- ○ Yes, I consent to having my survey answers paired with my facebook activity
- ○ No I do not consent

Schools

What college do you attend?

Agnes Scott College
Appalachian State University
Arizona State University
Baldwin Wallace College
Ball State University
Bard College
Bemidji State University
Berea College
Berry College
Bowdoin

What building do you live in?

- Appleton Hall
- Baxter House
- Burnett House
- Chamberlain Hall
- Coleman Hall
- Coles Tower
- Heimreich House
- Howard Hall
- Howell House
- Hyde Hall
- Ladd House
- MacMillan House
- Maine Hall
- Moore Hall
- Osher Hall
- Quinby House

Demographics

How long have you been a student at your school? This is my:

- 1st year
- 2nd year
- 3rd year
- 4th year
- 5th year/Continuing student

What is your gender?

- Male
- Female
- Other

With what ethnicity do you primarily identify?

- African/African-American
- Asian/Asian-American
- European/European-American
- Latina/o
- Native American
- Mixed Race
- Other (please specify) ________

What is your political orientation?

	Very Conservative			Neutral			Very Liberal
Political Orientation	O	O	O	O	O	O	O

Experience with the Competition

Is your school participating in "Conservation Nationals"?

- O Yes
- O No
- O I don't know

How often did you visit your school's Building Dashboard website during the competition?

Never	Once or twice	3 - 5 times	6 - 10 times	More than 10 times
O	O	O	O	O

Please indicate to what extent you agree or disagree with each of the following statements, using the scale provided.

	Strongly Disagree	Disagree	Mildly Disagree	Neither Agree Nor Disagree	Mildly Agree	Agree	Strongly Agree
I felt empowered by the competition.	O	O	O	O	O	O	O
I felt motivated to compete.	O	O	O	O	O	O	O
Others at my school were motivated to compete.	O	O	O	O	O	O	O
The competition motivated me to use	O	O	O	O	O	O	O

less electricity.							
The competition motivated me to use less water.	○	○	○	○	○	○	○
I was interested in how my dorm was doing relative to other dorms on my campus.	○	○	○	○	○	○	○
I was interested in how my school was doing relative to other schools.	○	○	○	○	○	○	○
The fact that other schools were competing at the same time made me feel like the competition on my campus was part of something larger.	○	○	○	○	○	○	○

Do you live in a residence hall or house that participated in the competition?

- ○ Yes
- ○ No
- ○ I don't know

Please answer the following questions to the best of your ability.

	0% - 10%	10% - 50%	50% - 90%	90% - 100%
What percentage of the students in competing residence halls do you think were aware of the competition?	○	○	○	○
What percentage of students in				

competing residence halls do you think actually changed their behavior as a result of the competition?	O	O	O	O

When visiting the Building Dashboard website, how interesting did you find each of the following features and applications?

	Not Interesting At All	Uninteresting	Mildly Uninteresting	Neutral	Mildly Interesting	Interesting	Ve[illegible] Intere[illegible]
The graph showing resource use in my dorm	O	O	O	O	O	O	O
The capacity to choose different units for expressing resource use (e.g. kWh, CO2, and $)	O	O	O	O	O	O	O
"Commit to Conserve" (where you commit to specific actions	O	O	O	O	O	O	O
"Competition Tweets" (where you view one or more Twitter feeds)	O	O	O	O	O	O	O
Display of current competition standing of dorms on	O	O	O	O	O	O	O

my campus							
Display of current competition standing between schools	○	○	○	○	○	○	○

How easily do you think others on your campus were able to find and use the following features on the dashboard?

	With Great Difficulty	With Difficulty	With Some Difficulty	Neutral	Somewhat Easily	Easily	Very Easily
Your campus's home page	○	○	○	○	○	○	○
Dorm's home page	○	○	○	○	○	○	○
Competition results page	○	○	○	○	○	○	○
Commitment to conserve feature	○	○	○	○	○	○	○
Discussion feature	○	○	○	○	○	○	○

Which of the following actions did you take during the competition that you would not have otherwise taken?

- ☐ Used a desk lamp instead of an overhead light
- ☐ Used a powerstrip to completely shut off power to unused computers, stereos, and other appliances when not in use
- ☐ Used natural daylight whenever possible (especially from 11 AM to 1 PM)
- ☐ Adjusted my computer's power setting so it would go to standby after 5 minutes of inactivity
- ☐ Turned off the water while I soaped up
- ☐ Took showers less frequently
- ☐ Turned off faucets when I brushed my teeth or shaved
- ☐ Waited to do laundry until I had a full load

- ☐ Air-dried laundry instead of using electric dryers
- ☐ Checked for leaking faucets and reported to facilities
- ☐ Took the stairs instead of the elevator
- ☐ Talked to hall mates about what they could do to conserve resources
- ☐ Turned off lights in hallways, bathrooms, and lounges at night.
- ☐ Convinced friends to visit the Building Dashboard website and make commitments
- ☐ Unplugged appliances that were not being used to eliminate phantom loads
- ☐ Visited the Building Dashboard daily to check on my dorm's resource use
- ☐ Got rid of a mini-fridge or shared with a neighbor
- ☐ Got more involved in student initiatives to reduce campus-wide resource use
- ☐ Kept windows closed when air-conditioning or heat was on
- ☐ Learned more about my college's policy on resource conservation
- ☐ Turned off lights in unoccupied public spaces when I saw them on
- ☐ Used my bicycle to get around campus
- ☐ Washed my clothes in cold water
- ☐ Recycled appropriate items (plastic, paper, glass, cardboard)
- ☐ Took showers that were 5 minutes or less
- ☐ Went tray-less in the dining hall

Perspectives on Changing Resource Use

The following groups at my school are trying to decrease energy consumption on campus.

	Strongly Disagree	Disagree	Slightly Disagree	Neither Agree nor Disagree	Slightly Agree	Agree	Strongly Agree
Undergraduate students	○	○	○	○	○	○	○
People living in my dorm	○	○	○	○	○	○	○
My friends	○	○	○	○	○	○	○

Please rate the following according to how much they motivate you to conserve

resources.

	Not Motivating	2	3	Neutral	5	6	Highly Motivating
My own well-being	O	O	O	O	O	O	O
My health	O	O	O	O	O	O	O
The well-being of other students on campus	O	O	O	O	O	O	O
Concern for the financial well-being of my college	O	O	O	O	O	O	O
Concern for the people	O	O	O	O	O	O	O
Concern for the animals	O	O	O	O	O	O	O
Concern for my country	O	O	O	O	O	O	O
Concern for future generations	O	O	O	O	O	O	O

What factors make it difficult for you to reduce your electricity consumption?

What factors make it difficult for you to reduce your water consumption?

What do you think are the benefits of reducing your water and electricity consumption?

Perspectives on Environment and Resource Use

Please indicate to what extent you agree or disagree with each of the following statements, using the scale provided.

	Strongly Disagree	Disagree	Slightly Disagree	Neither Agree nor Disagree	Slightly Agree	Agree	Strongly Agree
I often feel a strong connection to nature.	○	○	○	○	○	○	○
I think of nature as a family that I belong to.	○	○	○	○	○	○	○
I see myself as a part of the greater circle of life.	○	○	○	○	○	○	○
I feel that all living things in this world are connected, and I am a part of that.	○	○	○	○	○	○	○
Like the tree in the forest, I feel I belong to nature.	○	○	○	○	○	○	○

Please indicate to what extent you agree or disagree with each of the following statements, using the scale provided. Answer truthfully; don't worry about what you think you are "supposed" to answer.

	Strongly Disagree	Disagree	Slightly Disagree	Neither Agree nor Disagree	Slightly Agree	Agree	Strongly Agree
I often think about electricity consumption when I turn a light or appliance on or off.	○	○	○	○	○	○	○
I consciously make							

decisions to minimize my electricity use.	○	○	○	○	○	○	○
I consciously make decisions to minimize other people's electricity use.	○	○	○	○	○	○	○
I often think about water consumption when I am taking a shower, flushing the toilet, washing my hands, or using water in other ways.	○	○	○	○	○	○	○
I consciously make decisions to minimize the amount of water I use.	○	○	○	○	○	○	○
I consciously make decisions to minimize the amount of water other people use.	○	○	○	○	○	○	○

Please indicate to what extent you agree or disagree with each of the following statements, using the scale provided. Answer truthfully; don't worry about what you think you are "supposed" to answer.

	Strongly Disagree	Disagree	Slightly Disagree	Neither Agree nor Disagree	Slightly Agree	Agree	Strongly Agree
I feel that I have relatively little ability to influence electricity consumption in my dorm.	○	○	○	○	○	○	○
I feel that I have relatively little ability							

to influence water consumption in my dorm.	○	○	○	○	○	○	○
My electricity use has an important impact on the environment.	○	○	○	○	○	○	○
My water use has an important impact on the environment.	○	○	○	○	○	○	○

Are you interested in participating in a US Green Building Council student group to further green building efforts on campusClick to write the question text

- ○ Yes
- ○ Maybe
- ○ No

Do we have your permission to share your email address with the USGBC so they can contact you?

- ○ yes
- ○ No

Powered by Qualtrics
